# Supplementary material for: Ecological interactions drive metabolomic diversification in Amazonian Pseudonocardia symbionts
Source: mSystems. 2026 Mar 27;11(4):e01810-25. doi: 10.1128/msystems.01810-25 (PMC13098279; doi:10.1128/msystems.01810-25)
Supplement: Supplemental material — Tables S1 to S3; Figures S1 to S28. [file msystems.01810-25-s0001.docx]

**Ecological Interactions Drive Metabolomic Diversification in Amazonian *Pseudonocardia* Symbionts**

**Authors**: Carlismari O. Grundmann^1,2^, Weilan G. P. Melo ^2,3^, Andrés M. Caraballo-Rodríguez^4^, Nina U. de A. Guardia^2^, Ivan L. F. Migliorini^2^, Ricardo R. Da Silva^2^, Pieter C. Dorrestein^4^, Norberto P. Lopes^2^, Cameron R. Currie^5^, Jon Clardy^6^, Mônica T. Pupo ^2*^

^1^Department of Chemistry and Biochemistry - University of California Santa Cruz (UCSC), Santa Cruz, CA, USA

^2^School of Pharmaceutical Sciences of Ribeirão Preto - University of São Paulo (FCFRP-USP), Ribeirão Preto, SP, Brazil

^3^Universidade Estadual da Região Tocantins do Maranhão (UEMASUL), Estreito, MA, Brazil

^4^Skaggs School of Pharmacy and Pharmaceutical Sciences - University of California San Diego (UCSD), San Diego, CA, USA

^5^Faculty of Health Sciences, McMaster University, Hamilton, ON, Canada

^6^Department of Biological Chemistry and Molecular Pharmacology, Harvard Medical School, Boston, MA, USA

* Corresponding author: [mtpupo@fcfrp.usp.br](mailto:mtpupo@fcfrp.usp.br)

# **Table S1.** Microbial strains isolated from attine ants collected in the Amazon region for metabolomic evaluation experiments. *Pseudonocardia* strains and *Escovopsis* ICBG 729 were isolated from *Paratrachymyrmex* ants, while *Escovopsis* ICBG 740 was isolated from *Acromyrmex* colonies.

| **ICBG Code** | **Microorganism** | **Collection Region** | **Geographic Coordinates** |
| --- | --- | --- | --- |
| **1025** | *Pseudonocardia* sp. | Anavilhanas, AM (Upper Region) - Population 2 | S2° 16' 13.7 W61° 01' 05.3 |
| **1034** | *Pseudonocardia* sp. | Anavilhanas, AM (Upper Region) - Population 2 | S2° 16' 13.7 W61° 01' 05.3 |
| **1050** | *Pseudonocardia* sp. | Anavilhanas, AM (Upper Region) - Population 2 | S2° 16' 16.9 W61° 01' 06.0 |
| **1102** | *Pseudonocardia* sp. | Anavilhanas, AM (Upper Region) - Population 2 | S2° 16' 13.7 W61° 01' 05.3 |
| **1111** | *Pseudonocardia* sp. | Anavilhanas, AM (Lower Region) - Population 1 | S2° 32.036 W60° 50.101 |
| **1122** | *Pseudonocardia* sp. | Reserva Ducke, AM - Population 3 | S2° 55.904 W59° 58.424 |
| **1123** | *Pseudonocardia* sp. | Anavilhanas, AM (Upper Region) - Population 2 | S2° 16' 13.7 W61° 01' 05.3 |
| **1124** | *Pseudonocardia* sp. | Anavilhanas, AM (Upper Region) - Population 2 | S2° 16' 13.7 W61° 01' 05.3 |
| **1125** | *Pseudonocardia* sp. | Anavilhanas, AM (Upper Region) - Population 2 | S2° 16' 13.7 W61° 01' 05.3 |
| **1127** | *Pseudonocardia* sp. | Anavilhanas, AM (Lower Region) - Population 1 | S2° 31' 23,4 W60° 49' 31,9 |
| **1138** | *Pseudonocardia* sp. | Anavilhanas, AM (Upper Region) - Population 2 | S2° 16' 13.7 W61° 01' 05.3 |
| **1140** | *Pseudonocardia* sp. | Anavilhanas, AM (Upper Region) - Population 2 | S2° 16' 17.2 W61° 01' 07.3 |
| **1143** | *Pseudonocardia* sp. | Anavilhanas, AM (Upper Region) - Population 2 | S2° 16' 13.7 W61° 01' 05.3 |
| **1144** | *Pseudonocardia* sp. | Anavilhanas, AM (Lower Region) - Population 1 | S2° 31.427 W60° 49.540 |
| **1145** | *Pseudonocardia* sp. | Anavilhanas, AM (Upper Region) - Population 2 | S2° 16' 13.7 W61° 01' 05.3 |
| **1146** | *Pseudonocardia* sp. | Anavilhanas, AM (Lower Region) - Population 1 | S2° 31.422 W60° 49.552 |
| **1229** | *Pseudonocardia* sp. | Anavilhanas, AM (Lower Region) - Population 1 | S2° 31.961 W60° 49.649 |
| **1230** | *Pseudonocardia* sp. | Reserva Ducke, AM - Population 3 | S2° 55.904 W59° 58.424 |
| **1231** | *Pseudonocardia* sp. | Anavilhanas, AM (Lower Region) - Population 1 | S2° 31' 23,4 W60° 49' 31,9 |
| **1239** | *Pseudonocardia* sp. | Anavilhanas, AM (Fazenda Vermelho) - Population 4 | S2° 35.9 W61° 1.50 |
| **1243** | *Pseudonocardia* sp. | Anavilhanas, AM (Lower Region) - Population 1 | S2° 31.434 W60° 49.527 |
| **1245** | *Pseudonocardia* sp. | Anavilhanas, AM (Lower Region) - Population 1 | S2° 31' 23,4 W60° 49' 31,9 |
| **1247** | *Pseudonocardia* sp. | Reserva Ducke, AM - Population 3 | S2° 55.904 W59° 58.424 |
| **1248** | *Pseudonocardia* sp. | Anavilhanas, AM (Upper Region) - Population 2 | S2° 16' 13.7 W61° 01' 05.3 |
| **1250** | *Pseudonocardia* sp. | Anavilhanas, AM (Upper Region) - Population 2 | S2° 16.242 W61° 01.113 |
| **1251** | *Pseudonocardia* sp. | Reserva Ducke, AM - Population 3 | S2° 55.808 W59° 58.479 |
| **1288** | *Pseudonocardia* sp. | Anavilhanas, AM (Upper Region) - Population 2 | S2° 16' 13.7 W61° 01' 05.3 |
| **1289** | *Pseudonocardia* sp. | Reserva Ducke, AM - Population 3 | S2° 55.904 W59° 58.424 |
| **1290** | *Pseudonocardia* sp. | Anavilhanas, AM (Upper Region) - Population 2 | S2° 16' 13.7 W61° 01' 05.3 |
| **1292** | *Pseudonocardia* sp. | Anavilhanas, AM (Upper Region) - Population 2 | S2° 16' 13.7 W61° 01' 05.3 |
| **1294** | *Pseudonocardia* sp. | Anavilhanas, AM (Upper Region) - Population 2 | S2° 16' 13.7 W61° 01' 05.3 |
| **1297** | *Pseudonocardia* sp. | Reserva Ducke, AM - Population 3 | S2° 55.904 W59° 58.424 |
| **1299** | *Pseudonocardia* sp. | Reserva Ducke, AM - Population 3 | S2° 55.825 W59° 58.529 |
| **1327** | *Pseudonocardia* sp. | Anavilhanas, AM (Upper Region) - Population 2 | S2° 16' 13.7 W61° 01' 05.3 |
| **1857** | *Pseudonocardia* sp. | Anavilhanas, AM (base) - Population 1 | S2° 31.447 W60° 49.473 |
| **1860** | *Pseudonocardia* sp. | Anavilhanas, AM (Fazenda Vermelho) - Population 4 | S2° 34.818 W61° 02.045 |
| **729** | *Escovopsis* | Anavilhanas, AM (Upper Region) - Population 2 | S2° 16' 13.7 W61° 01' 05.3 |
| **740** | *Escovopsis* | Anavilhanas, AM (Lower Region) - Population 1 | S2° 32.036 W60° 50.101 |

^
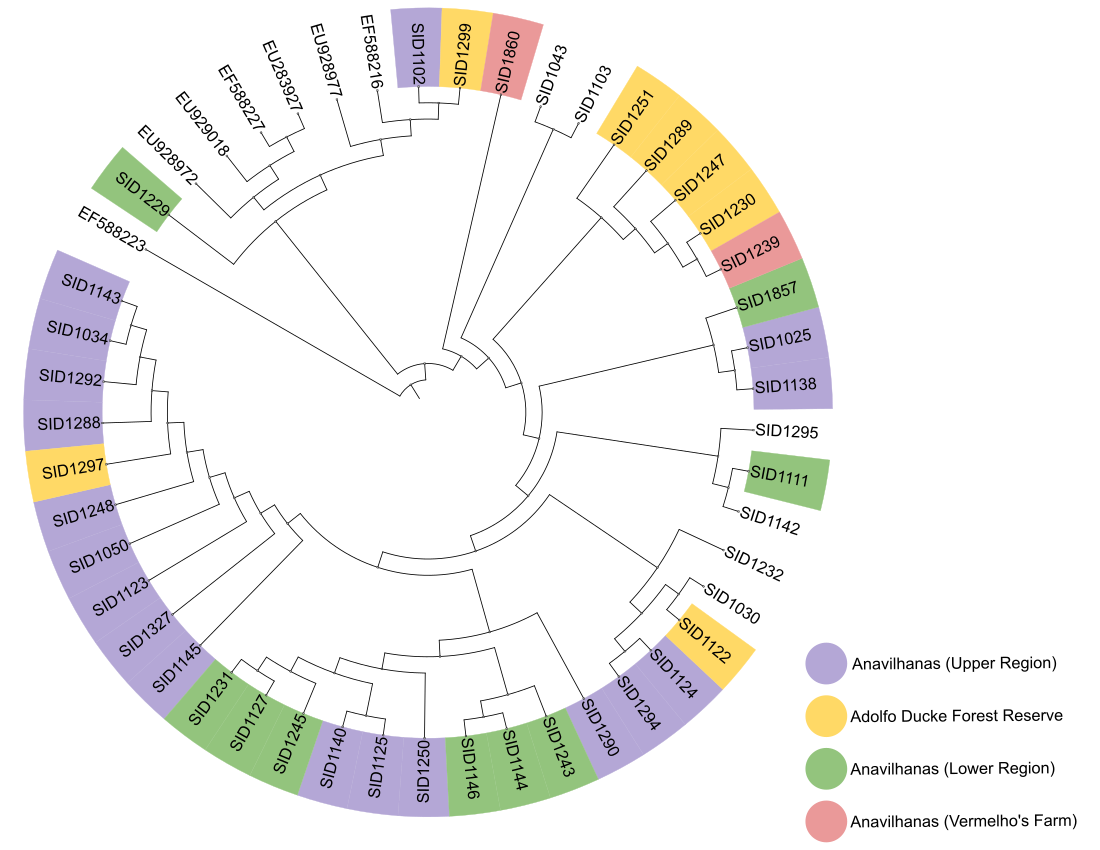
^

# **Figure S1.** Phylogenetic analysis of *Pseudonocardia* strains based on 16S rRNA gene sequences. The tree was constructed using the Maximum Likelihood method with 1,000 bootstrap replicates and rooted with *Streptomyces griseus* (EF588223) as the outgroup. Strains analyzed in this study are color-coded according to their collection site, as indicated in the legend. Strains beginning with the prefix “SID” belong to the laboratory’s in-house culture collection, whereas the remaining sequences correspond to reference strains obtained from the literature.

**
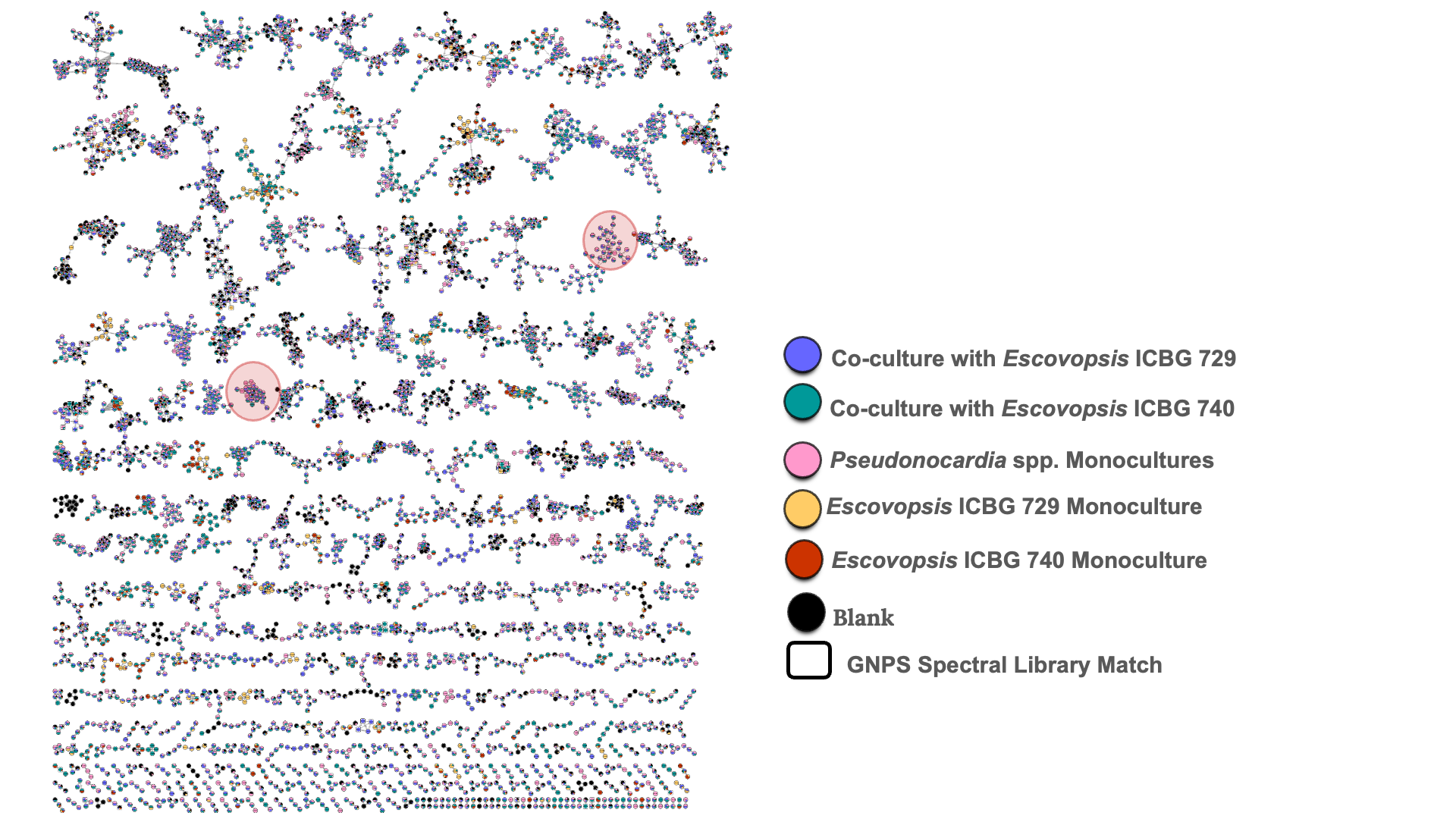
**

**Figure S2.** Overview of the molecular networks constructed from LC-MS/MS data of 36 *Pseudonocardia* strains cultured in monoculture and in co-culture with *Escovopsis* ICBG 729 and ICBG 740. Nodes represent MS/MS spectra, with edges connecting structurally related molecules based on spectral similarity. Node colors correspond to sample types as indicated in the legend, node sizes reflect the number of MS/MS spectra (scans), and pie charts indicate the relative abundance of each feature in the different culture conditions. The two clusters highlighted with red circles correspond exclusively to metabolites from *Pseudonocardia* sp. ICBG 1122, either in monoculture or in co-culture. Self-loops are not shown.


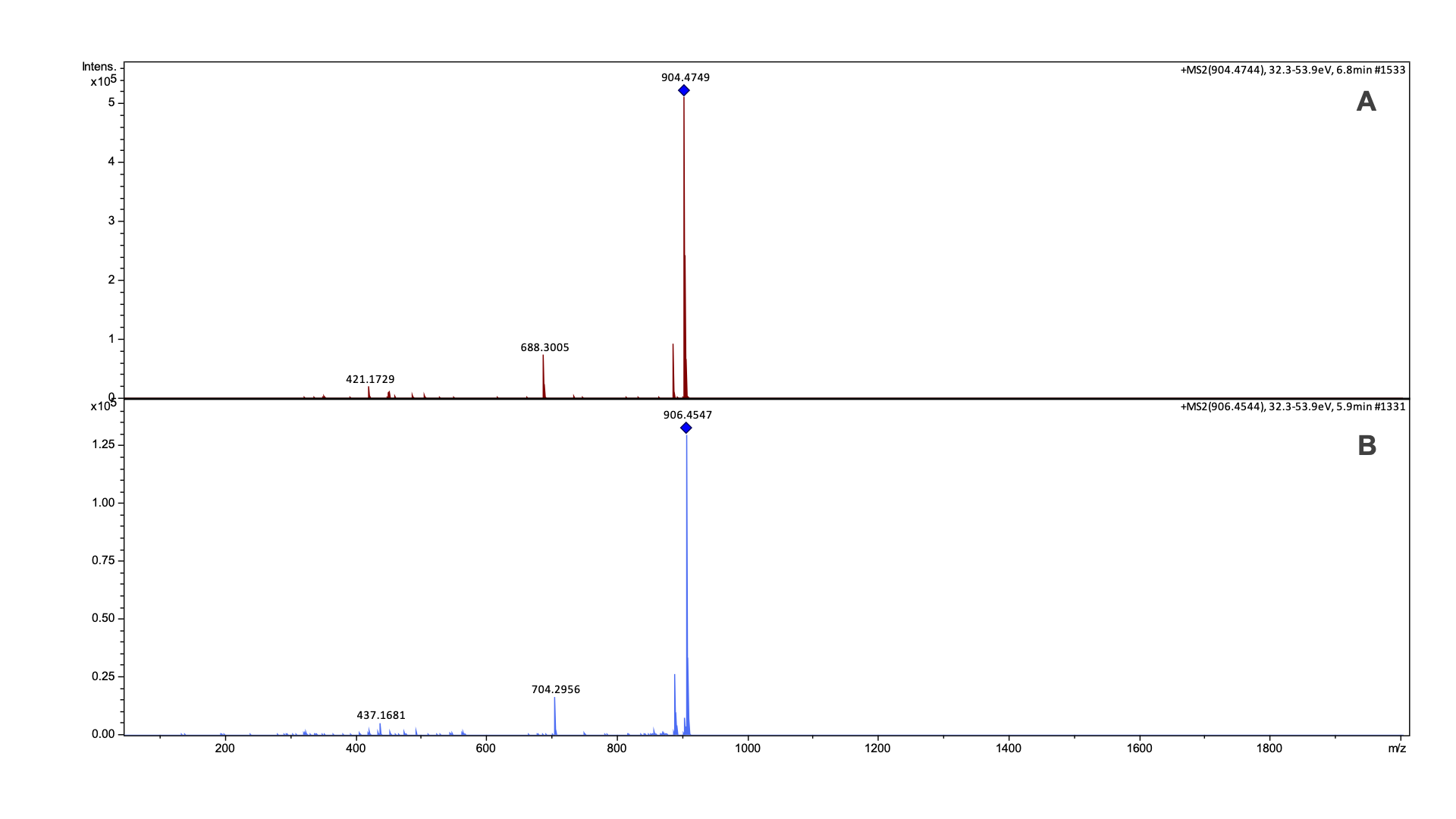


# **Figure S3.** Comparison of HR-ESI-MS/MS spectra of the [M+Na]⁺ ions at *m/z* 904.4749 **(A)** and 906.4547 **(B)**, corresponding to compounds **(1)** and **(2)**, respectively, identified in *Pseudonocardia* sp. ICBG 1122. For **(1)** *m/z* calculated is 904.4756 and error is 0.5 ppm. For **(2)** *m/z* calculated is 906.4573, and error is 2.8 ppm.


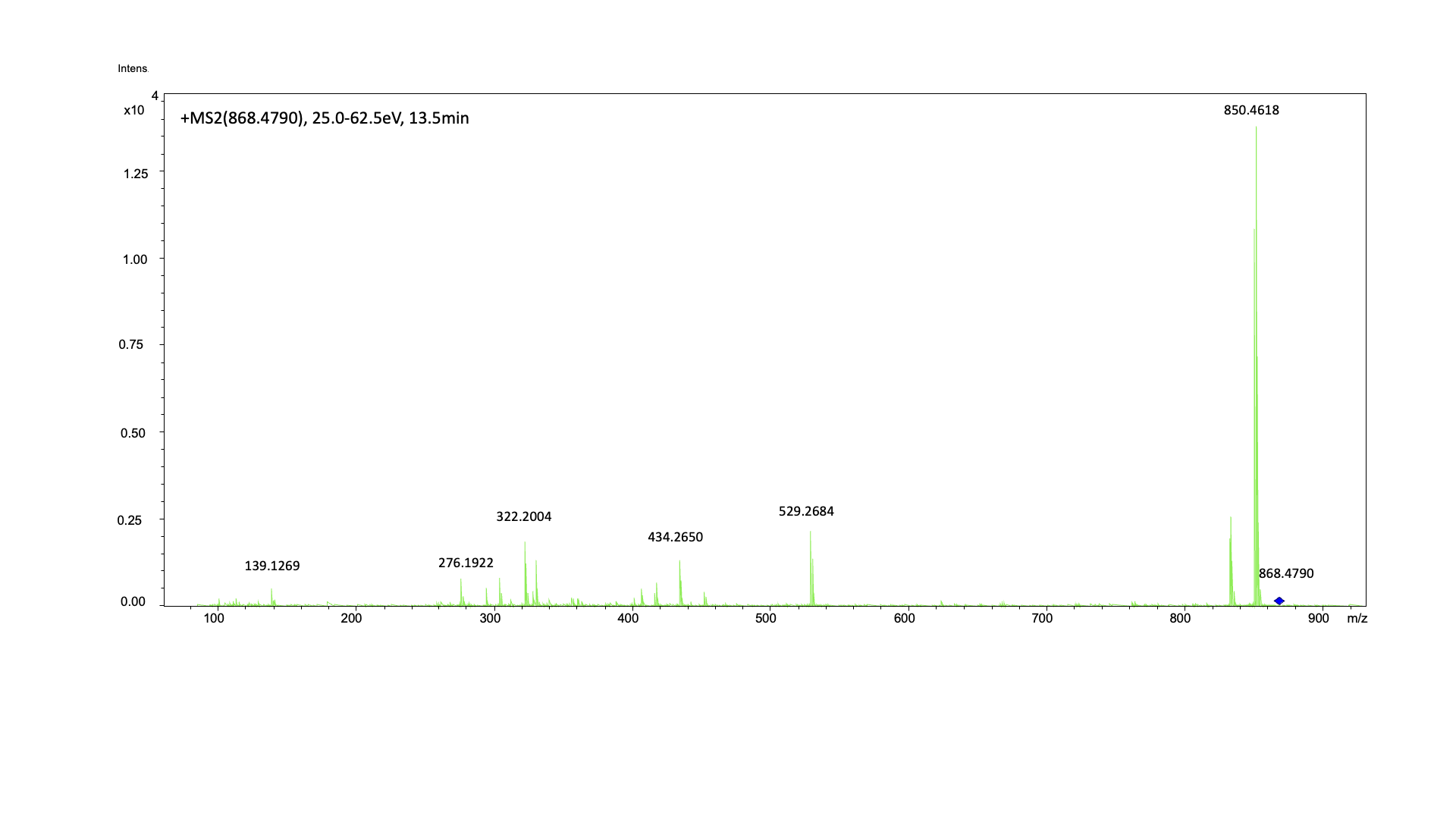


# **Figure S4.** HR-ESI-MS/MS spectrum of the [M+H]⁺ ion at *m/z* 868.4790 corresponding to compound **(3)** identified in *Pseudonocardia* sp. ICBG 1122. For **(3)** *m/z* calculated is 850.4669 and error is -5.9 ppm.


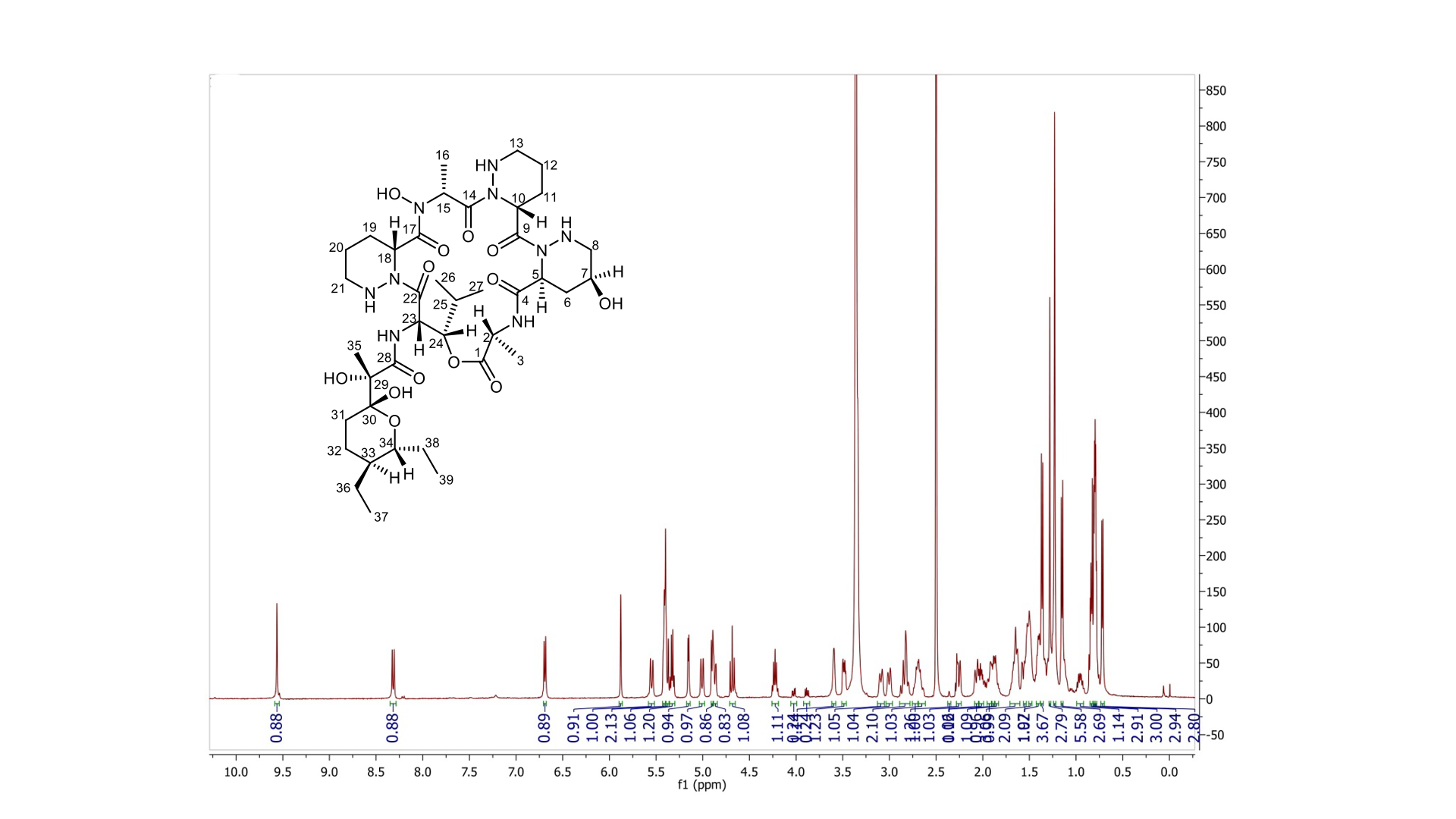
**Figure S5.** ^1^H NMR spectrum of dentigerumycin F **(3)** (DMSO-d_6_, 500 MHz).

# **Table S2.** Comparison of the ¹H NMR chemical shifts and integrals reported in the literature for dentigerumycin F (1) with those observed in isolated fraction from the *Pseudonocardia* ICBG 1122 extract.

| **Dentigerumycin F**  **(Literature - 500 MHz, DMSO-d_6_ )** | | | **Isolated Fraction**  **(500 MHz, DMSO-d_6_)** | | |
| --- | --- | --- | --- | --- | --- |
| **Position** | **δH (J in Hz)** | **Integral** | **Position** | **δH (J in Hz)** | **Integral** |
| **2** | 4.22, m | 1 | 2 | 4.23, m | 1 |
| **2-NH** | 6.69, d (8.5) | 1 | 2-NH | 6.69, d (8.5) | 1 |
| **3** | 1.10, d (7.5) | 3 | 3 | 1.15, d (7.34) | 3 |
| **5** | 4.90, d (7.5, 1.5) | 1 | 5 | 4.90, d (7.36) | 1 |
| **6a** | 2.26, br d (14.0) | 1 | 6a | 2.26, b d (16.5) | 1 |
| **6b** | 1.88, m | 1 | 6b | 1.86, m | 1 |
| **7** | 3.60, br s | 1 | 7 | 3.59, br s | 1 |
| **7-OH** | 5.12, d (5.0) | 1 | 7-OH | 5.15, d (4.74) | 1 |
| **8** | 2.84, m | 2 | 8 | 2.84, m | 2 |
| **8-NH** | 4.85, dd (12.0, 2.0) | 1 | 8-NH | 4,87, m | 1 |
| **10** | 5.42, dd (6.5, 3.0) | 1 | 10 | 5.41, m | 1 |
| **11a** | 1.92, m | 1 | 11a | 1.91, m | 1 |
| **11b** | 1.85, m | 1 | 11b | 1.85, m | 1 |
| **12** | 1.51, m | 2 | 12 | 1.51, m | 2 |
| **13a** | 3.09, br d (13.0) | 1 | 13a | 3.09, br d (12.05) | 1 |
| **13b** | 2.70, m | 1 | 13b | 2.71, m | 1 |
| **13-NH** | 5.01, d (13.0) | 1 | 13-NH | 5.01, d (12.84) | 1 |
| **15** | 5.34, q (7.5) | 1 | 15 | 5.33, q (6.93) | 1 |
| **15-N-OH** | 9,56, s | 1 | 15-N-OH | 9.56, s | 1 |
| **16** | 1.37, d (7.5) | 3 | 16 | 1.36, d (7.40) | 3 |
| **18** | 5.40, dd (5.5, 1.5) | 1 | 18 | 5.40, m | 1 |
| **19a** | 2.07, br d (12.0) | 1 | 19a | 2.05, m | 1 |
| **19b** | 1.66, m | 1 | 19b | 1.67, m | 1 |
| **20** | 1.50, m | 2 | 20 | 1.50, m | 2 |
| **21a** | 3.00, br. d (12.0) | 1 | 21a | 3.00, br d (12.61) | 1 |
| **21b** | 2.69, m | 1 | 21b | 2.69, m | 1 |
| **21-NH** | 5.54, d (12.5, 1.5) | 1 | 21-NH | 5.55, d (12.8) | 1 |
| **23** | 4.69, dd (11.5, 11.0) | 1 | 23 | 4,.68, t (10.97) | 1 |
| **23-NH** | 8.31, d (11.0) | 1 | 23-NH | 8.32, d (10,9) | 1 |
| **24** | 5.37, dd (11.5, 1,5) | 1 | 24 | 5.37, m | 1 |
| **25** | 2.03, m | 1 | 25 | 2.01, m | 1 |
| **26** | 0.80, d (6.5) | 3 | 26 | 0.79, t (overlaid) | 3 |
| **27** | 0.72, d (6.5) | 3 | 27 | 0.72, d (6.81) | 3 |
| **29-OH** | 5.37, s | 1 | 29-OH | 5.37, br s | 1 |
| **30-OH** | 5.88, s | 1 | 30-OH | 5.88, s | 1 |
| **31a** | 1.65, m | 1 | 31a | 1.65, m | 1 |
| **31b** | 1.55, m | 1 | 31b | 1.54, m | 1 |
| **32** | 1.64, m | 1 | 32 | 1.63, m | 1 |
| **33** | 1.13, m | 1 | 33 | not observed | |
| **34** | 3.49, m | 1 | 34 | 3.48, m | 1 |
| **35** | 1.29, s | 3 | 35 | 1.28, s | 3 |
| **36a** | 1.38, m | 1 | 36a | 1.36, d (7.40) | 4 |
| **36b** | 0.96, m | 1 | 36b | 0.95, m | 1 |
| **37** | 0.84, t (6.5) | 3 | 37 | 0.84, m | 3 |
| **38** | 1.41, m | 2 | 38 | 1.41, m | 2 |
| **39** | 0.80, t (7.0) | 3 | 39 | 0.8, t (overlaid) | 3 |


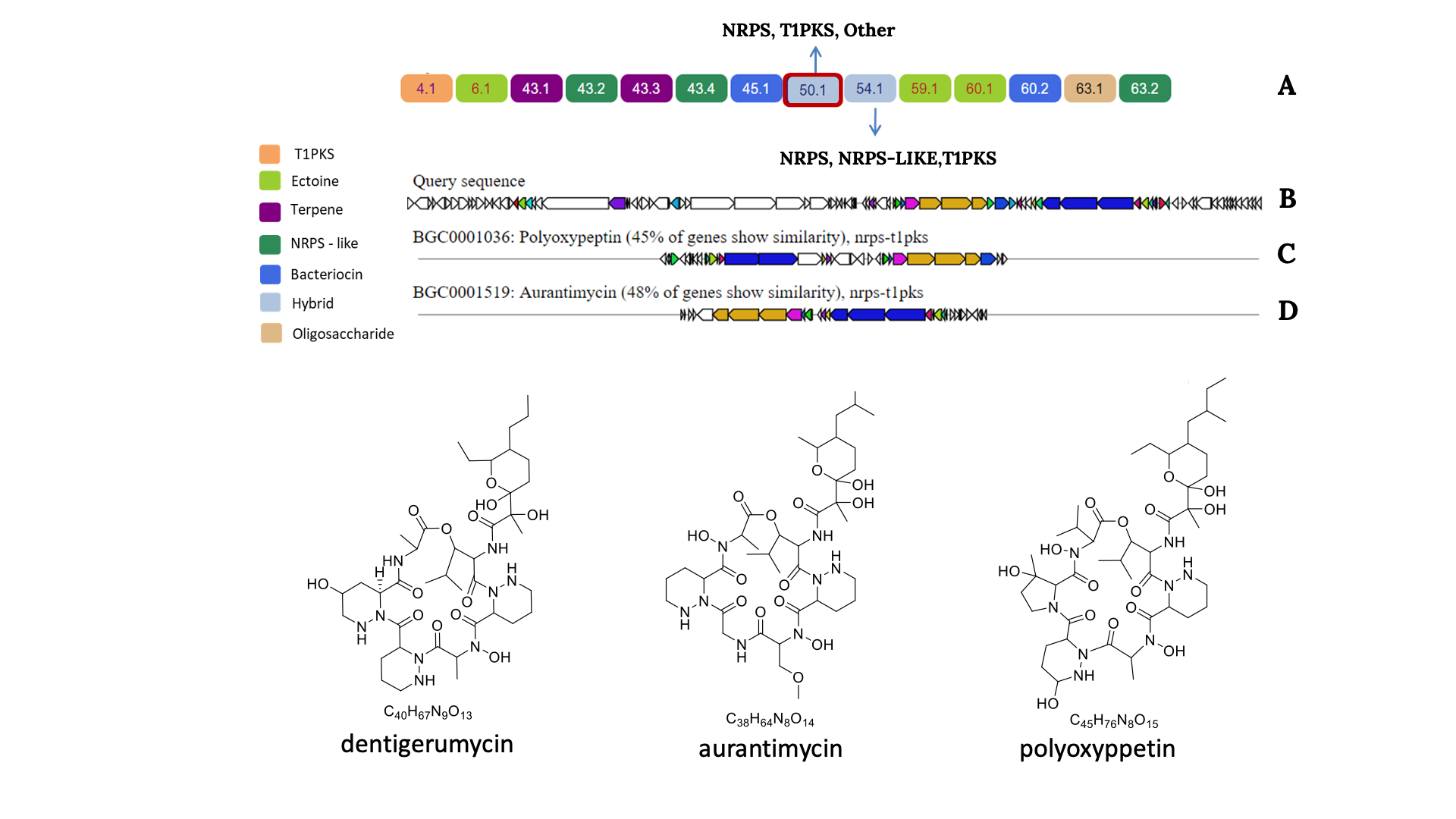
**Figure S6.** Biosynthetic gene clusters (BGCs) associated with the genome of *Pseudonocardia* sp. ICBG 1122. Highlighted are domains 50.1 and 54.1, which correspond to hybrid BGCs according to the legend **(A)**. Gene sequences linked to domain 50.1 **(B)** and their association with the production of polyoxypeptin **(C)** and aurantimycin **(D)** are shown. Matching colors across panels B, C, and D indicate putative homologous genes, as determined by significant BLAST hits identified through the antiSMASH platform.


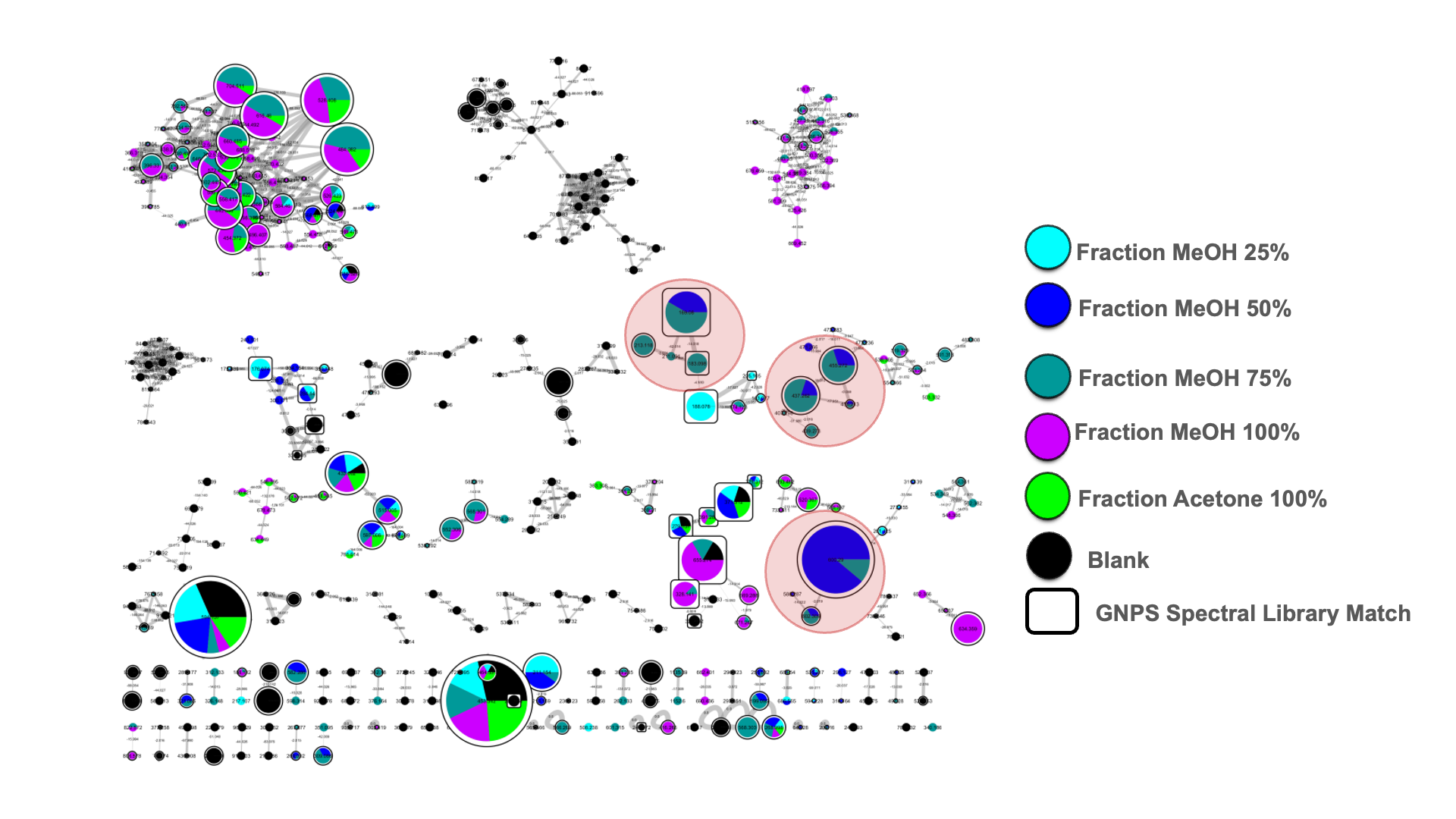


**Figure S7.** Molecular networks overview generated from LC-MS/MS data of methanol and SPE fractions obtained from small-scale fermentation of *Pseudonocardia* strain ICBG1025. Nodes represent MS/MS spectra, with edges connecting structurally related molecules based on spectral similarity. Node colors correspond to sample types as indicated in the legend, node sizes reflect the number of MS/MS spectra (scans), and pie charts indicate the relative abundance of each feature in the different methanolic fractions (25%, 50%, 75%, and 100%) and 100% acetone fraction. Black nodes represent ions detected in the blank control. Nodes outlined in white indicate GNPS library matches. Red circles highlight clusters enriched only with MeOH 50% and 75% fractions (blue and green, respectively).Node size is proportional to the number of MS/MS scans acquired for each ion.

**
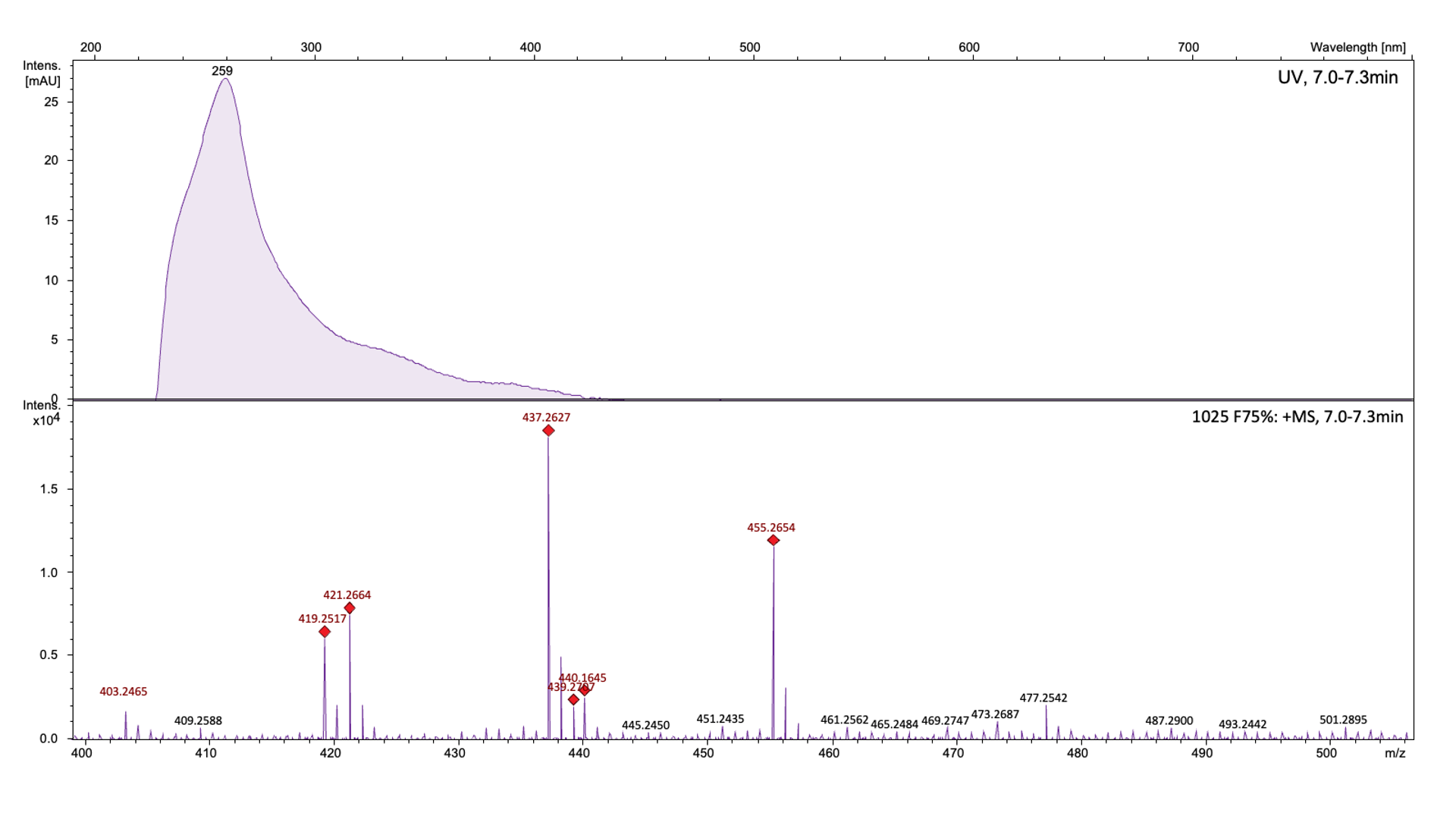
**

# **Figure S8.** HR-ESI-MS/MS and UV-Vis spectra of the [M+H]⁺ ion at *m/z* 455.2654 corresponding to compound **(6)** identified in the 75% MeOH *Pseudonocardia* sp. ICBG 1025 fraction. For **(6)** *m/z* calculated is 455.2614 and error is 8.7 ppm.

#


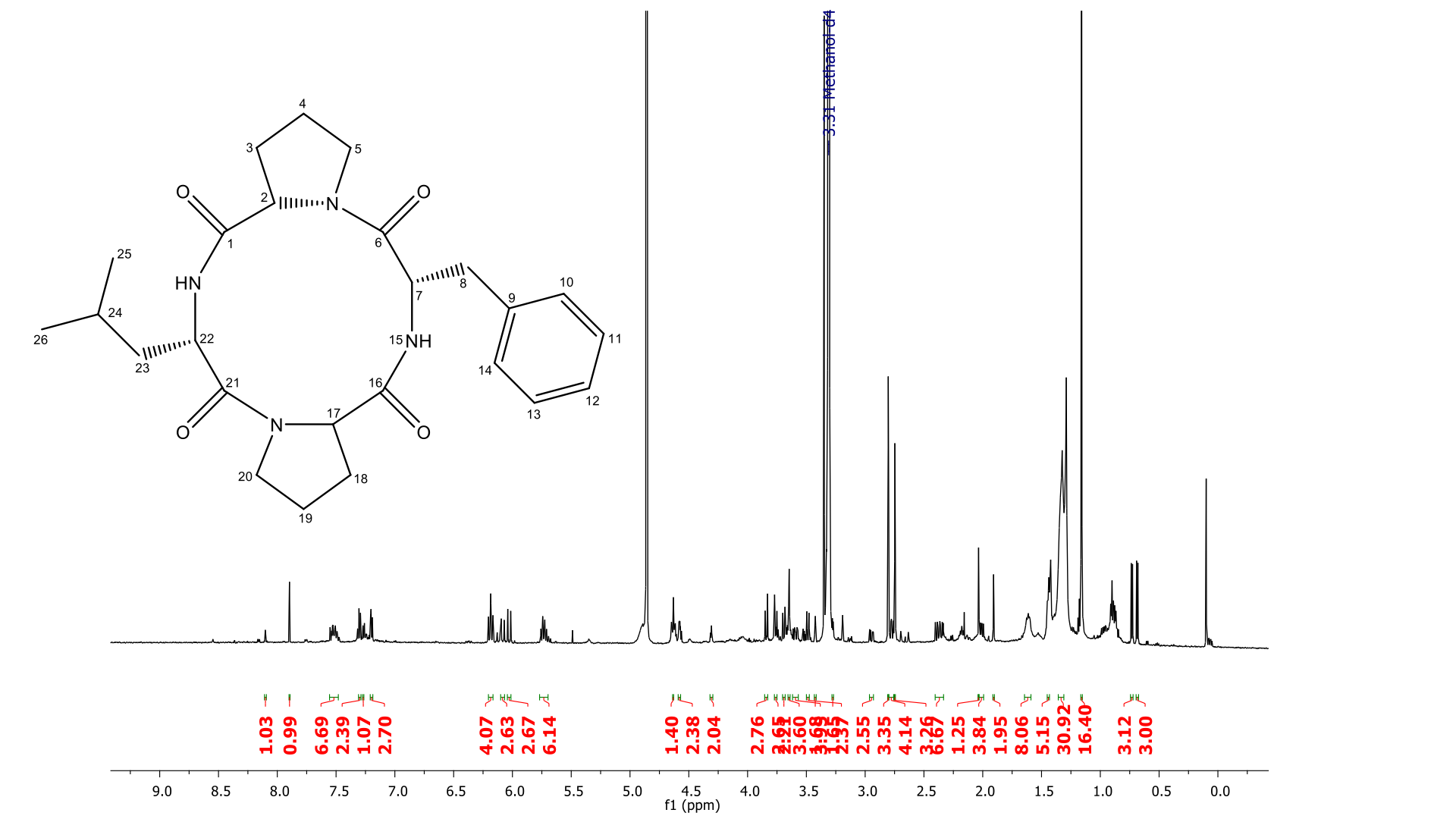


# **Figure S9.** ^1^H NMR spectrum of provipeptide A **(6)** (CDCl_3_, 600 MHz).


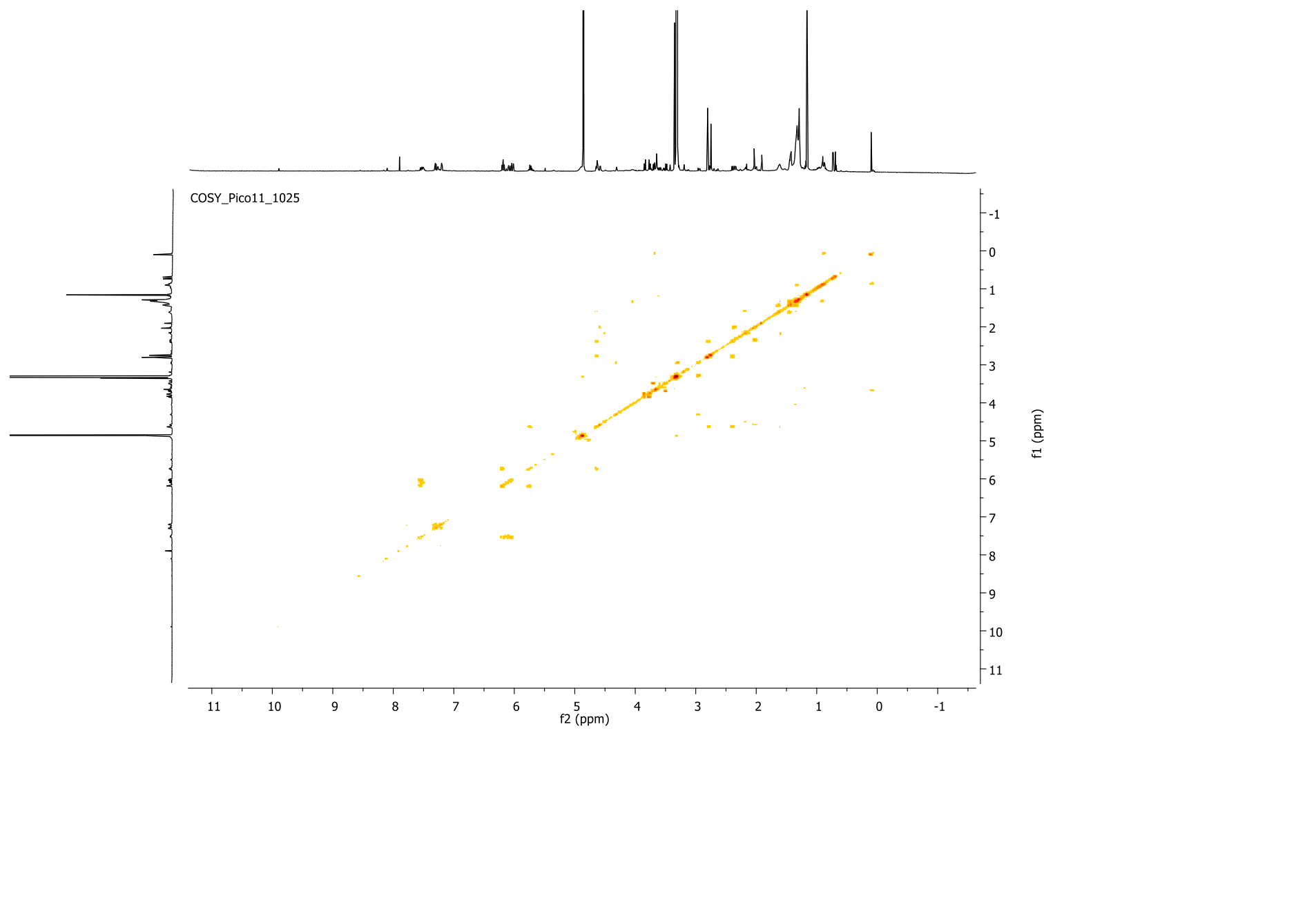
**Figure S10.** *g*COSY NMR spectrum of provipeptide A **(6)** (CDCl_3_, 600 MHz; ^13^C, 125 MHz).


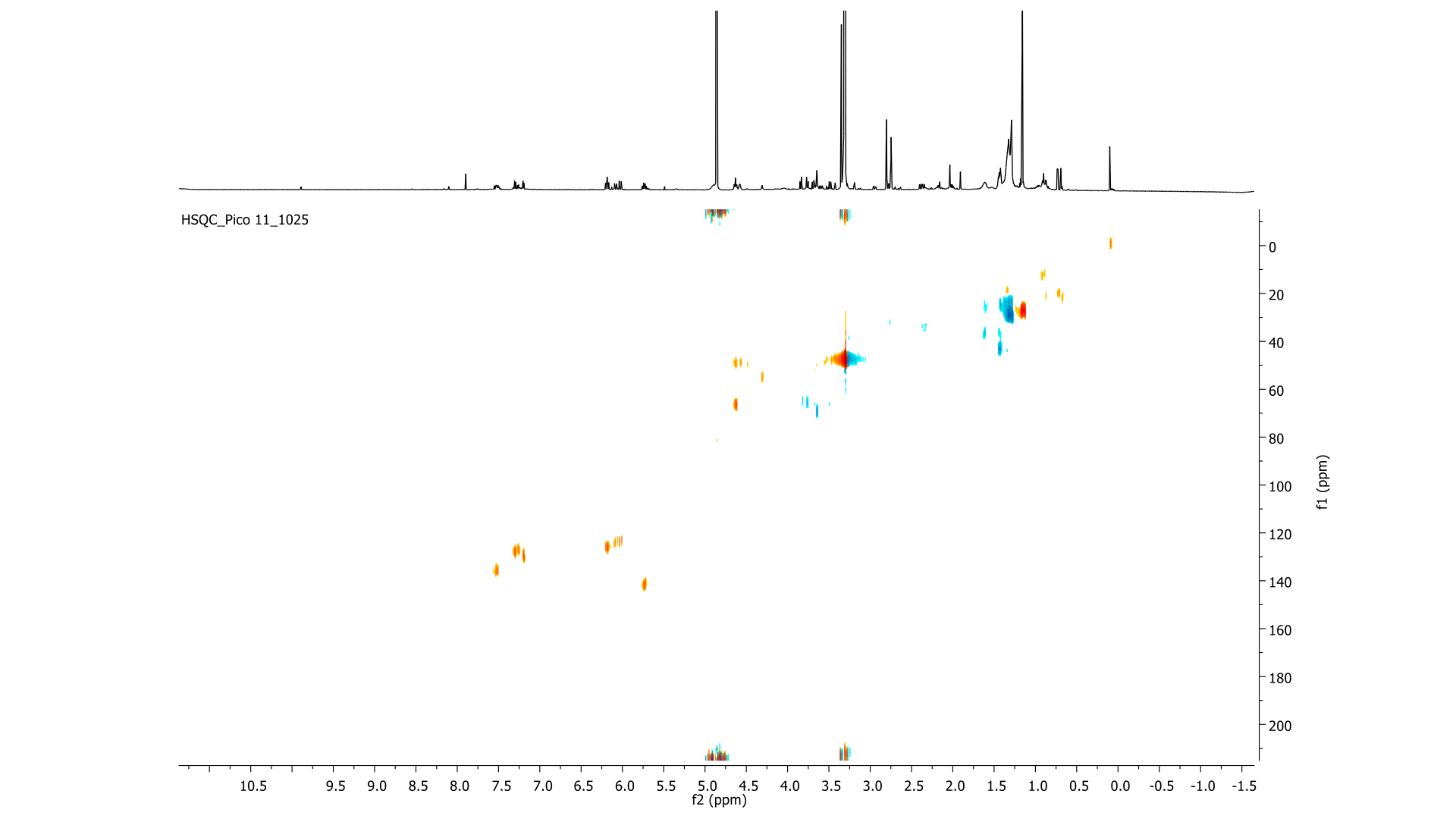


# **Figure S11.** *g*HSQC NMR spectrum of provipeptide A **(6)** (CDCl_3_, 600 MHz; ^13^C, 125 MHz).


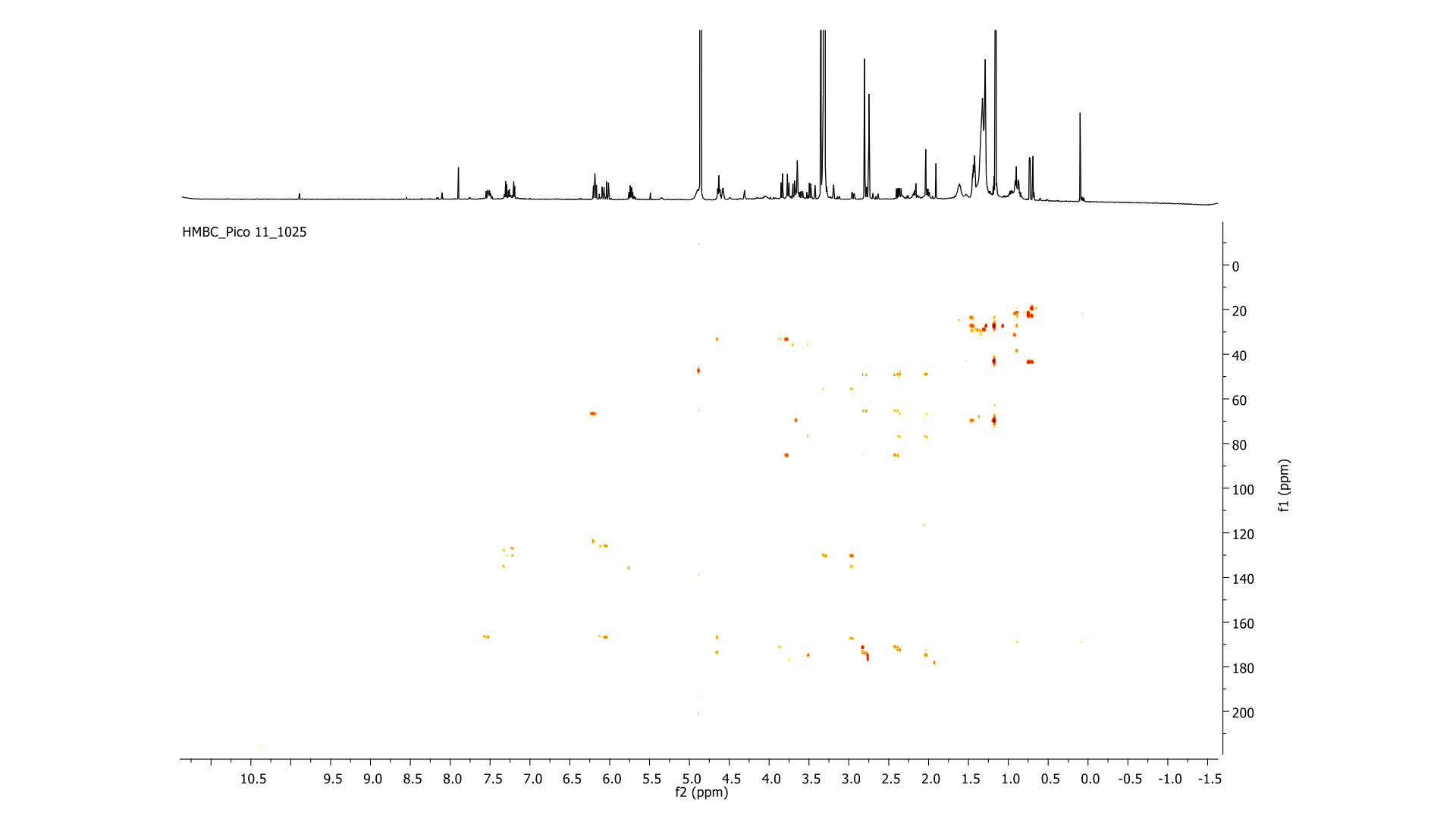


# **Figure S12.** *g*HMBC NMR spectrum of provipeptide A **(6)** (CDCl_3_, 600 MHz; ^13^C, 125 MHz).

# **Table S3.** 1D and 2D NMR data of provipeptide A **(6)** in CDCl_3_ isolated from *Pseudonocardia* ICBG 1025 extract (^1^H at 600 MHz and ^13^C at 125 MHz).

| **Residue** | **Position** | **δ H**  **(J in Hz)** | **Integral** | **δ C** | ***g*COSY** | ***g*HMBC** |
| --- | --- | --- | --- | --- | --- | --- |
| Proline 1 | **1** |  |  | 166.9 |  | C2, C3 |
|  | **2** | 4.63, dd | 1 | 66.6 | H3a, H3b | C1, C3 |
|  | **3a** | 2.03, m | 1 | 36.7 | H3b, H4a | C1, C2 |
|  | **3b** | 2.37, m | 1 |  | H3a, H4b | C1, C2, C4 |
|  | **4a** | 1.36, m | 1 | 26.8 | H4b | C3, C5 |
|  | **4b** | 2.04, m | 1 |  | H3b, H4a, H5a | C3, C5 |
|  | **5a** | 3.2, m | 1 | 47.4 | H5b, H4b | C4, C6 |
|  | **5b** | 3.43, m | 1 |  | H5a | C4, C6 |
| Phenylalanine | **6** |  |  | 167.3 |  | C5, C7, C8 |
|  | **7** | 4.31, dd | 1 | 55.0 | H8a, H8b | C6, C8, C9 |
|  | **8a** | 2.95, dd | 1 | 38.3 | H7, H8b | C6, C7, C9 |
|  | **8b** | 3.32, dd | 1 |  | H7, H8a | C6, C7, C9 |
|  | **9** |  |  | 135.2 |  | C7, C8, C19 |
|  | **10** | 7.19, m | 1 | 127.6 | H11 | C9, C11, C12 |
|  | **11** | 7.3, m | 1 | 127.3 | H10, H12 | C10, C12 |
|  | **12** | 7.2, m | 1 | 129.8 | H11, H13 | C11, C13, C10, C14 |
|  | **13** | 7.3, m | 1 | 127.3 | H12, H14 | C12, C14 |
|  | **14** | 7.19, m | 1 | 127.6 | H13 | C9, C12, C13 |
|  | **15 N-H** | ND |  |  |  |  |
| Proline 2 | **16** |  |  | 166.9 |  | C7, C17, C18 |
|  | **17** | 4.65, dd | 1 | 66.6 | H18a, H18b | C16, C18, C19 |
|  | **18a** | 2.39, m | 1 | 33.9 | H17, H18b, H19a | C16, C17, C19 |
|  | **18b** | 2.79, m | 1 |  | H18a, H19a | C17, C19 |
|  | **19a** | 1.68, m | 1 | 26.0 | H18b, H19b, H20a | C17, C18, C20 |
|  | **19b** | 2.03, m | 1 |  | H19a, H18b | C18, C20 |
|  | **20a** | 3.64, m | 1 | 48.2 | H20b, H19b | C19, C21 |
|  | **20b** | 3.73, m | 1 |  | H20a, H19b | C18, C19 |
| Leucine | **21** |  |  | 173.7 |  | C20, C22, C23 |
|  | **22** | 4.58, m | 1 | 48,9 | 23b | C1, C21, C23 |
|  | **23a** | 1.62, m | 1 | 37.5 | H22, H23b, H24 | C21, C22, C24 |
|  | **23b** | 2.2, m | 1 |  | H22, H23a | C21, C22, C24 |
|  | **24** | 1.43, m | 1 | 24.6 | H25, H26 | C23, C25, C26 |
|  | **25** | 0.69, d | 1 | 22.3 | H24 | C23, C24, C26 |
|  | **26** | 0.73, d | 1 | 20.2 | H24 | C23, C24, C25 |
|  | **27 N-H** | 7.9, s | 1 |  | H22 | C2 |


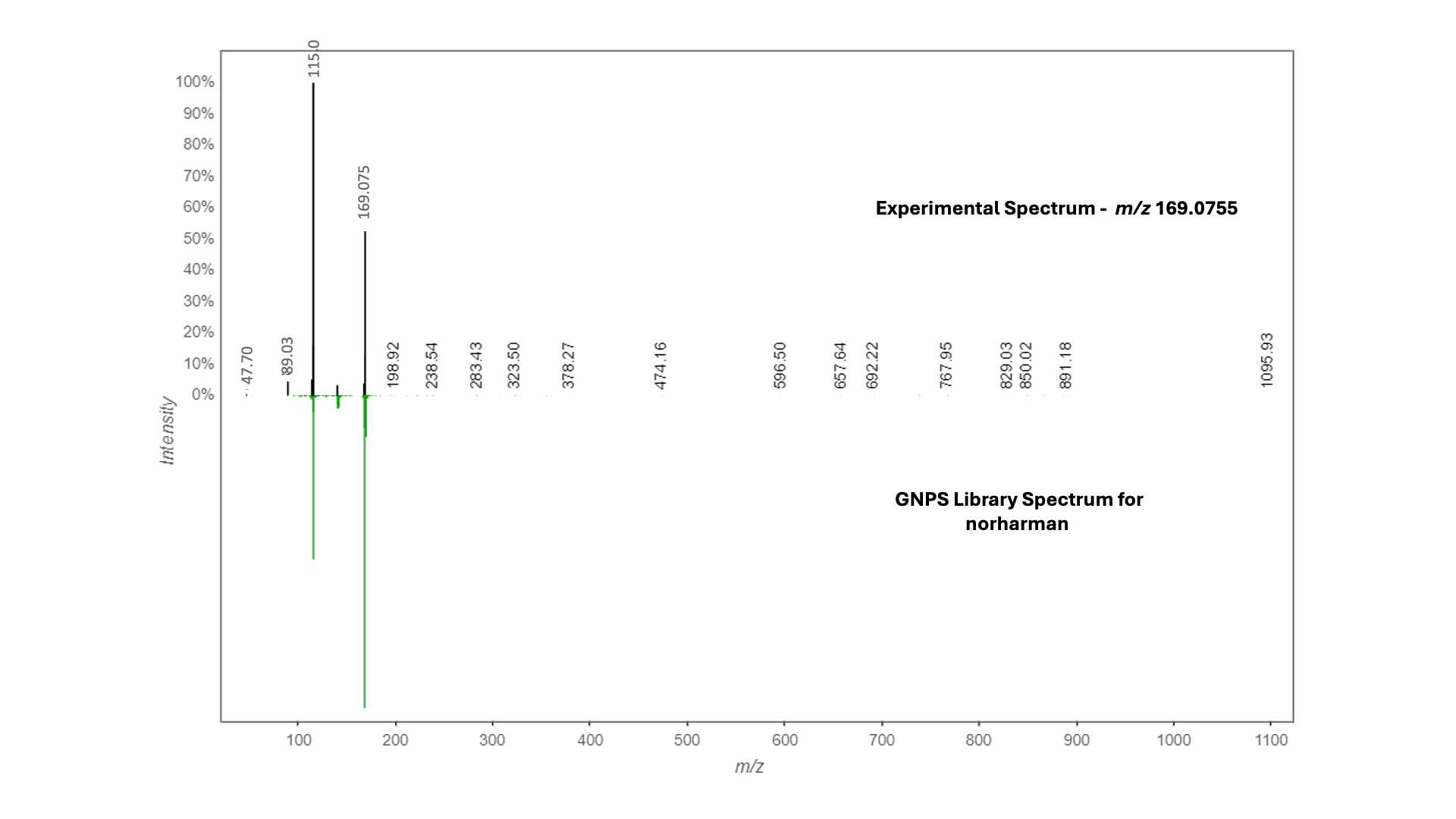
**Figure S13.** GNPS mirror match plot for norharman **(4)**. Green peaks represent the MS/MS library spectrum, while black peaks correspond to the MS/MS spectrum related to the ion *m/z* 169.0755 acquired from the microbial sample extract *Pseudonocardia* sp. ICBG 1025. The comparison has a cosine score of 0.94. For **(4)** *m/z* calculated is 169.0760 and error is 3.0 ppm.

#


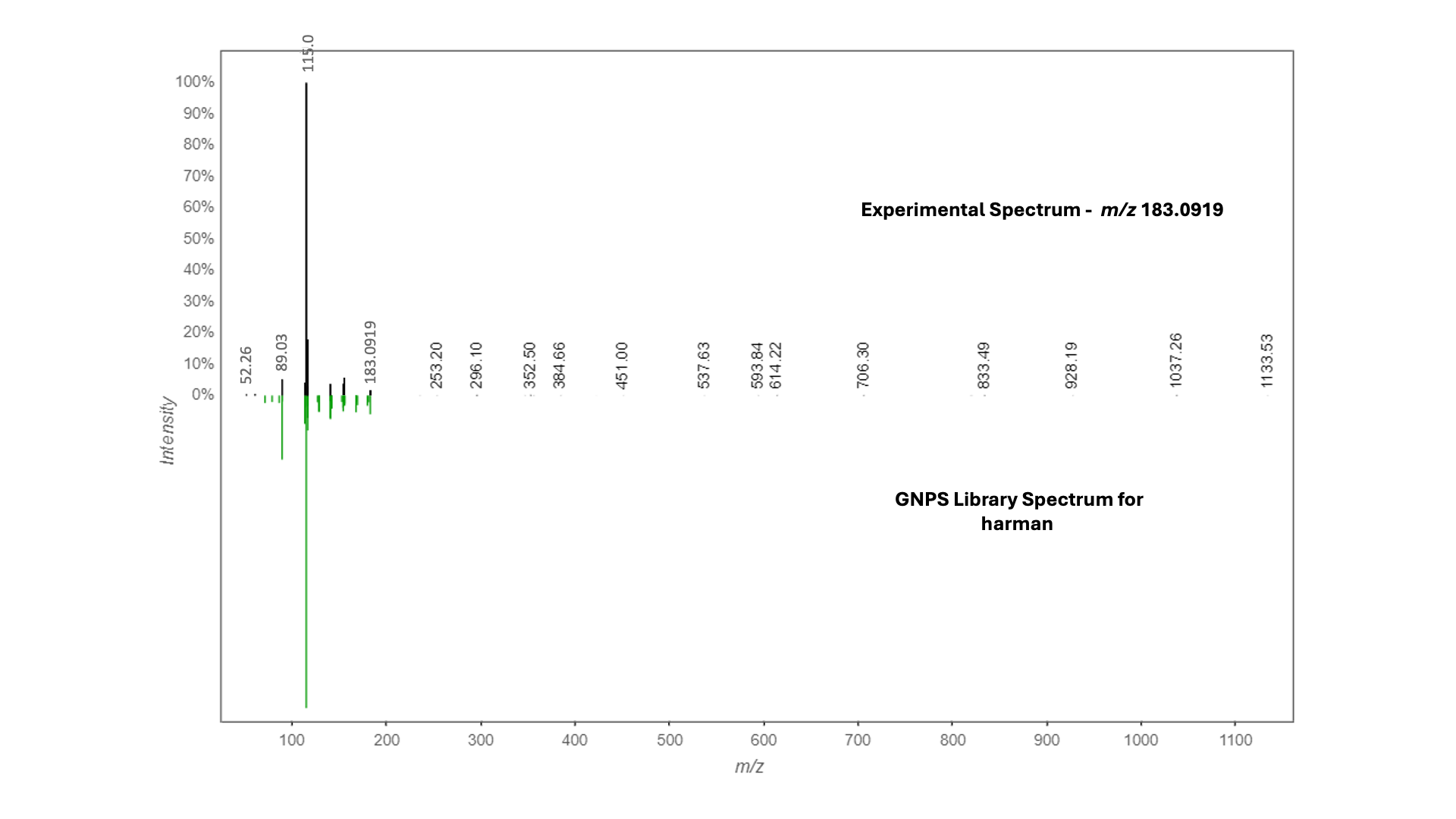


# **Figure S14.** GNPS mirror match plot for harman **(5)**. Green peaks represent the MS/MS library spectrum, while black peaks correspond to the MS/MS spectrum related to the ion *m/z* 183.0919 acquired from the microbial sample extract *Pseudonocardia* sp. ICBG 1025. The comparison has a cosine score of 0.95. For **(5)** *m/z* calculated is 183.0917 and error is 2.0 ppm.


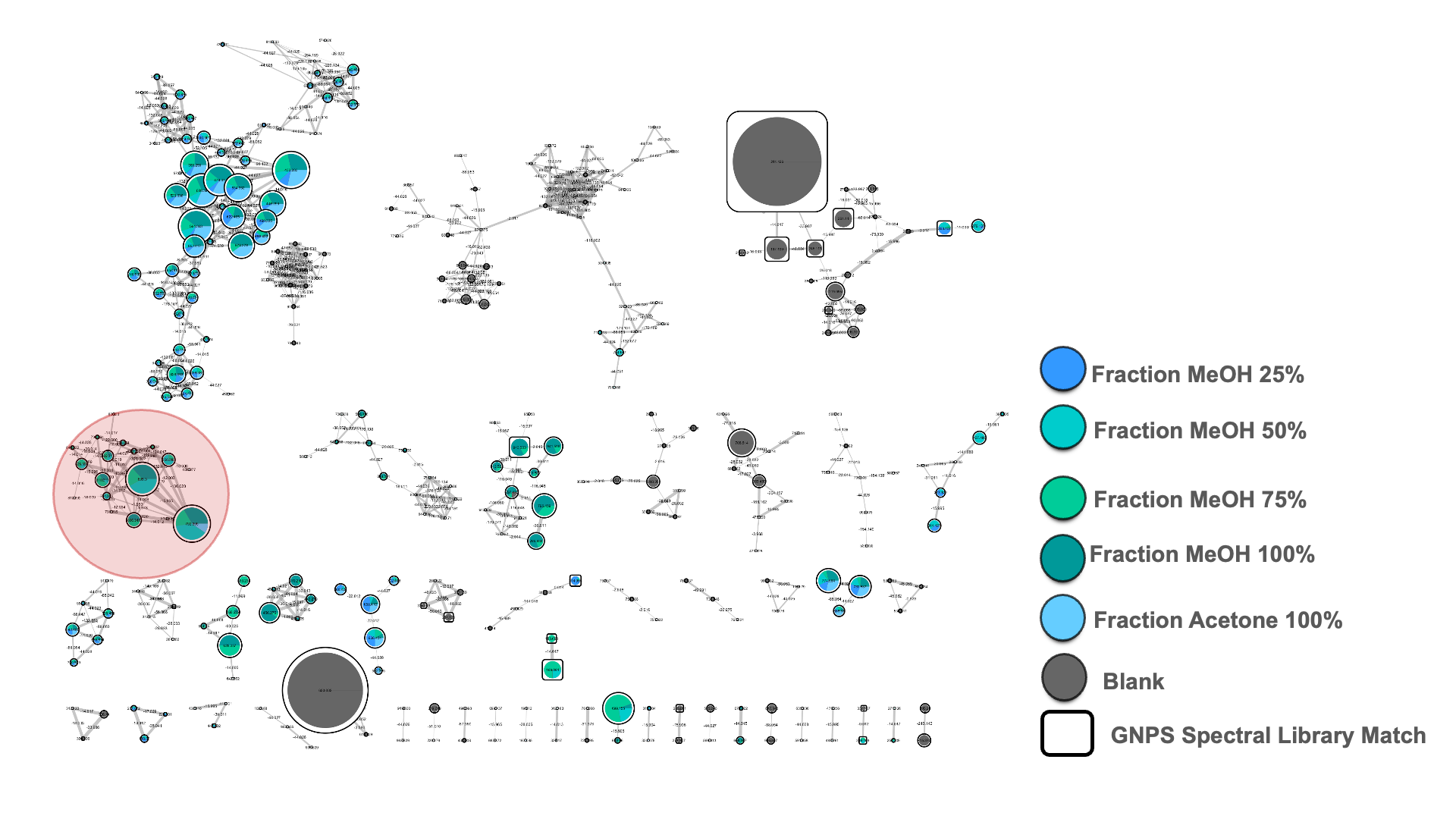


**Figure S15 -** Overview of all ions detected in the LC-MS/MS analysis of small-scale cultures of *Pseudonocardia* sp. ICBG 1860, grouped by molecular similarity using GNPS molecular networking. Nodes represent MS/MS spectra, with edges connecting structurally related molecules based on spectral similarity. Node colors correspond to sample types as indicated in the legend, node sizes reflect the number of MS/MS spectra (scans), and pie charts indicate the relative abundance of each feature in the different methanolic fractions (25%, 50%, 75%, and 100%) and 100% acetone fraction. Gray nodes represent blank samples. A molecular cluster enriched in methanolic fractions 50%, 75% and 100% is highlighted in red.The node sizes in the network are proportional to the number of MS/MS scans associated with each ion.


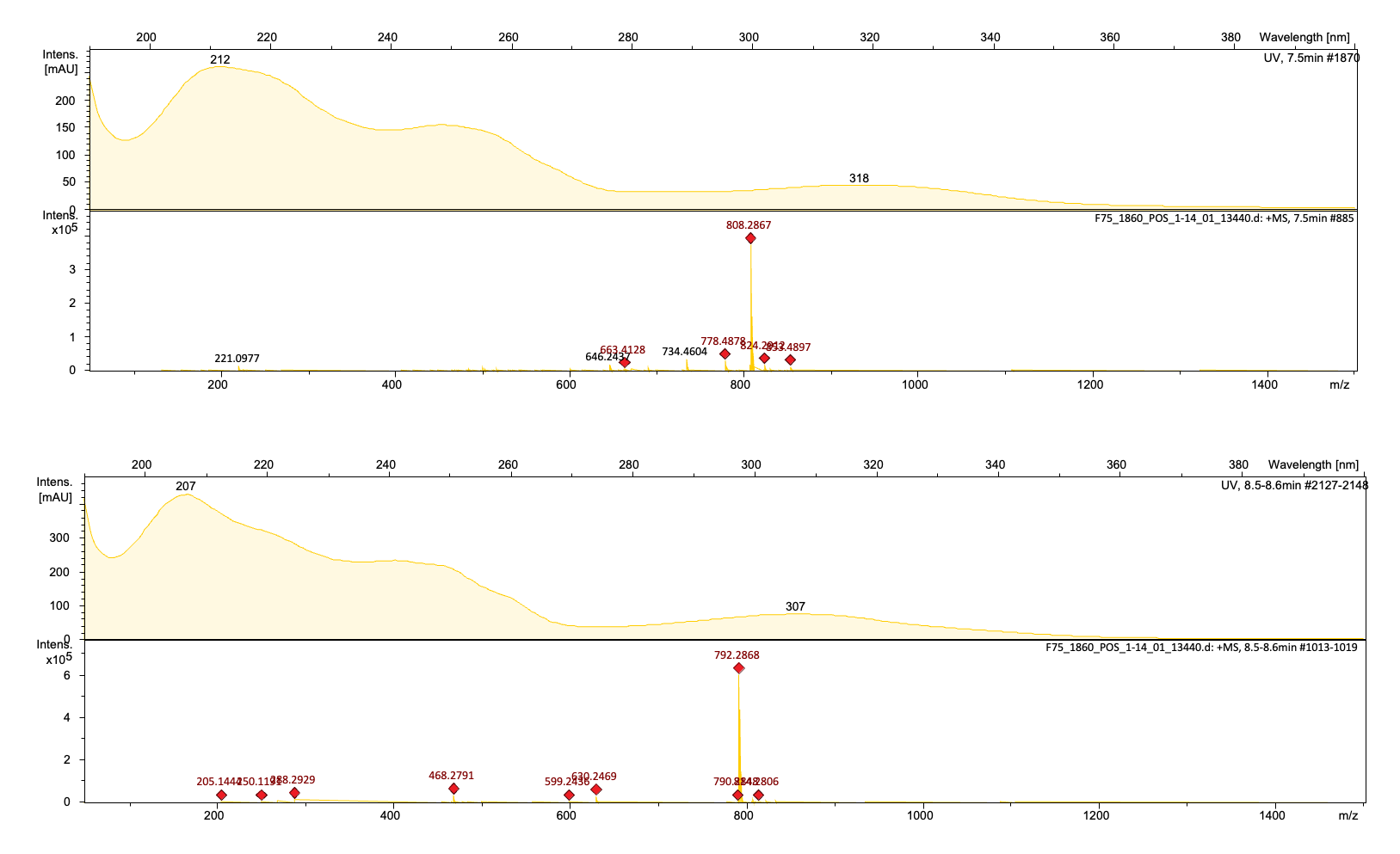


**Figure S16.** HR-ESI-MS and UV-Vis spectra of the [M+H]⁺ ion at *m/z* 792.2868 corresponding to compound **(7)** and its putative new analog at *m/z* 808.2867, both identified in *Pseudonocardia* sp. ICBG 1860 fractions**.** For **(7)** *m/z* calculated is 792.2862 and error is 0.8 ppm. UV-visible absorption spectra was measured in ACN at 254 nm.


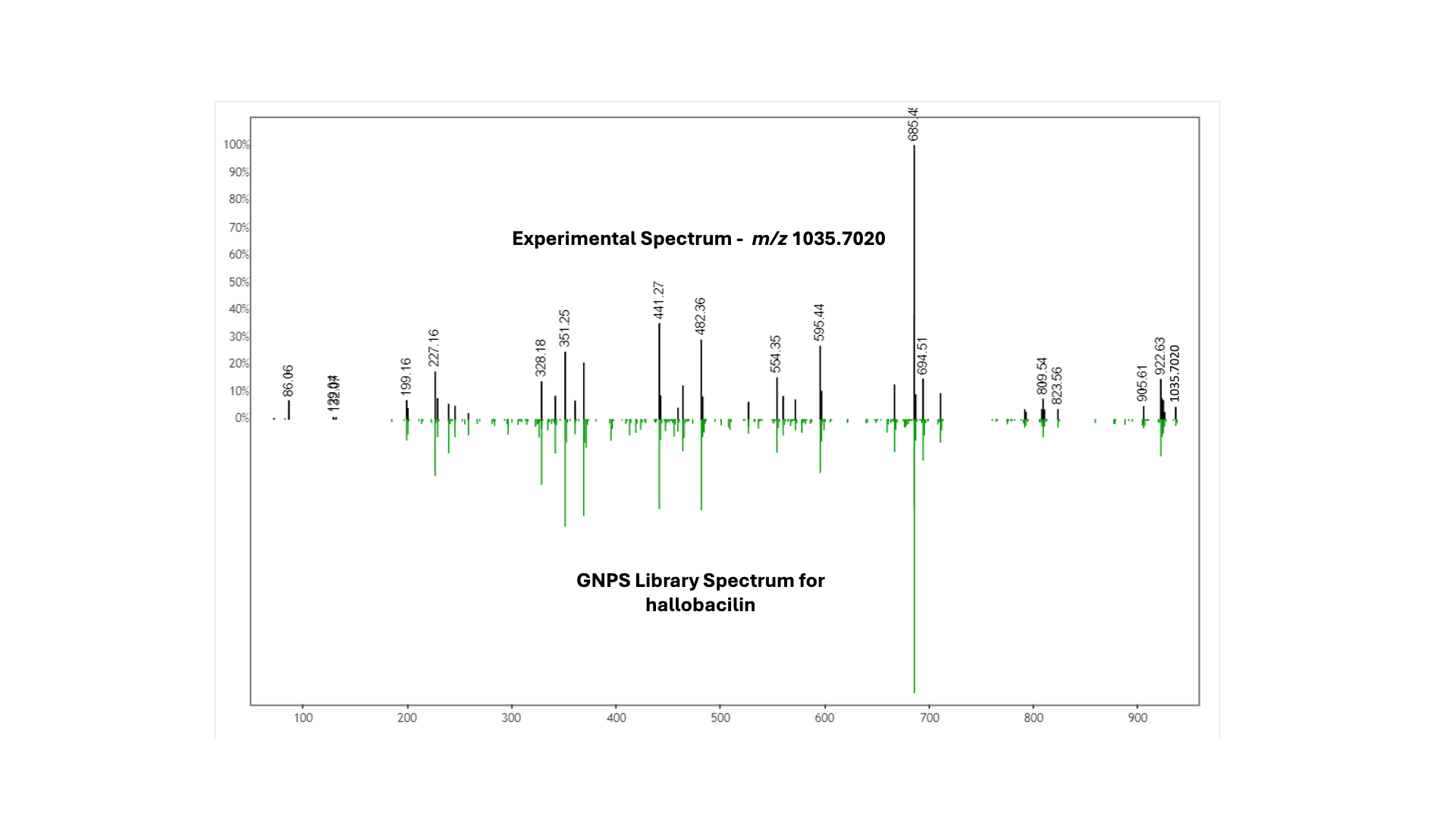
**Figure S17.** GNPS mirror match plot for hallobacilin **(8)**. Green peaks represent the MS/MS library spectrum, while black peaks correspond to the MS/MS spectrum related to the ion *m/z* 1035.7020 acquired from the co-culture sample extract *Pseudonocardia* sp. ICBG 1050 x *Escovopsis* sp. ICBG 729. The comparison has a cosine score of 0.90. For **(8)** *m/z* calculated is 1035.7064 and error is 4.2 ppm.


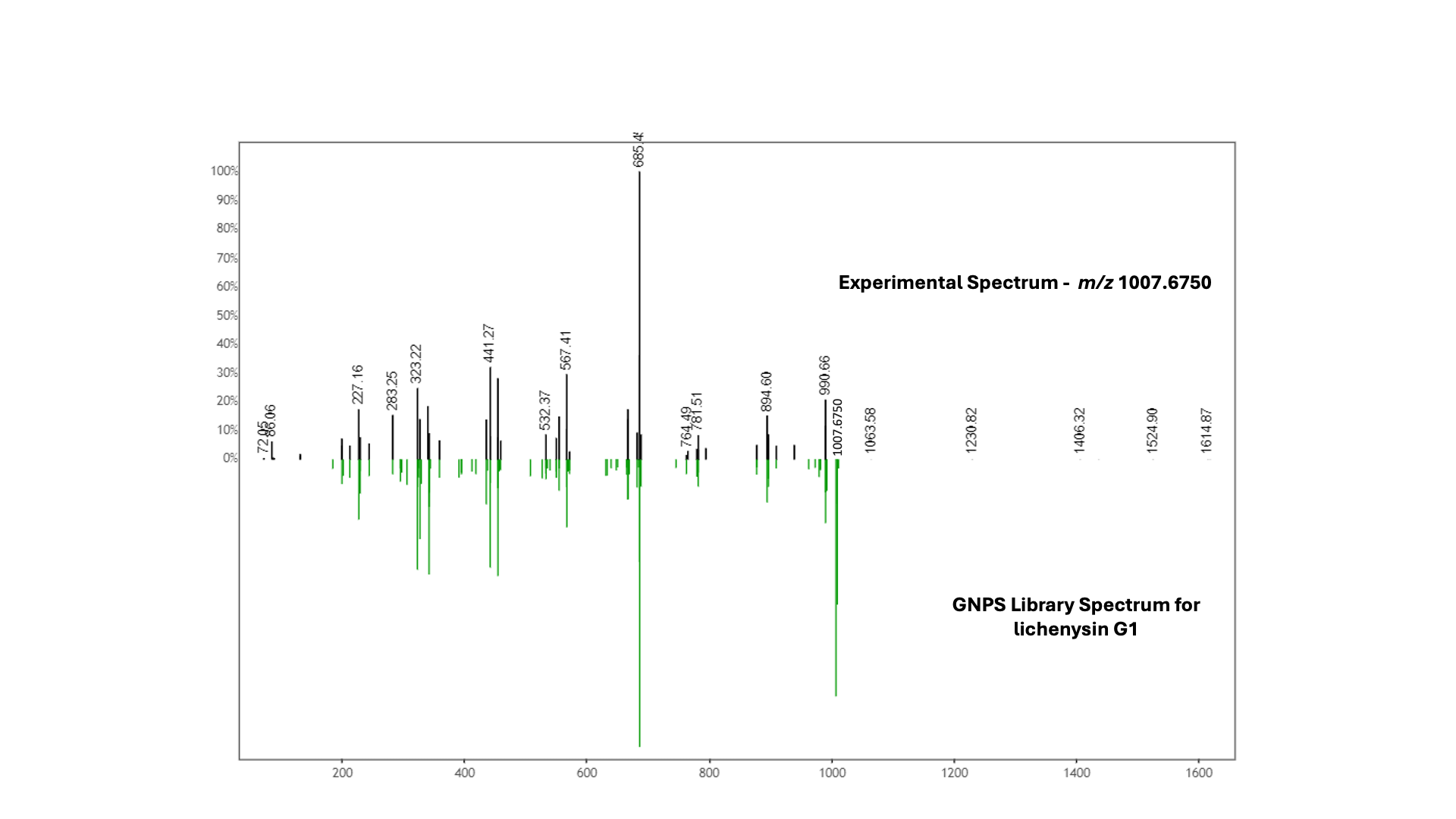
**Figure S18.** GNPS mirror match plot for lichenysin G1 **(9)**. Green peaks represent the MS/MS library spectrum, while black peaks correspond to the MS/MS spectrum related to the ion *m/z* 1007.6750 acquired from the co-culture sample extract *Pseudonocardia* sp. ICBG 1050 x *Escovopsis* sp. ICBG 729. The comparison has a cosine score of 0.89. For **(9)** *m/z* calculated is 1007.6712 and error is 3.8 ppm.


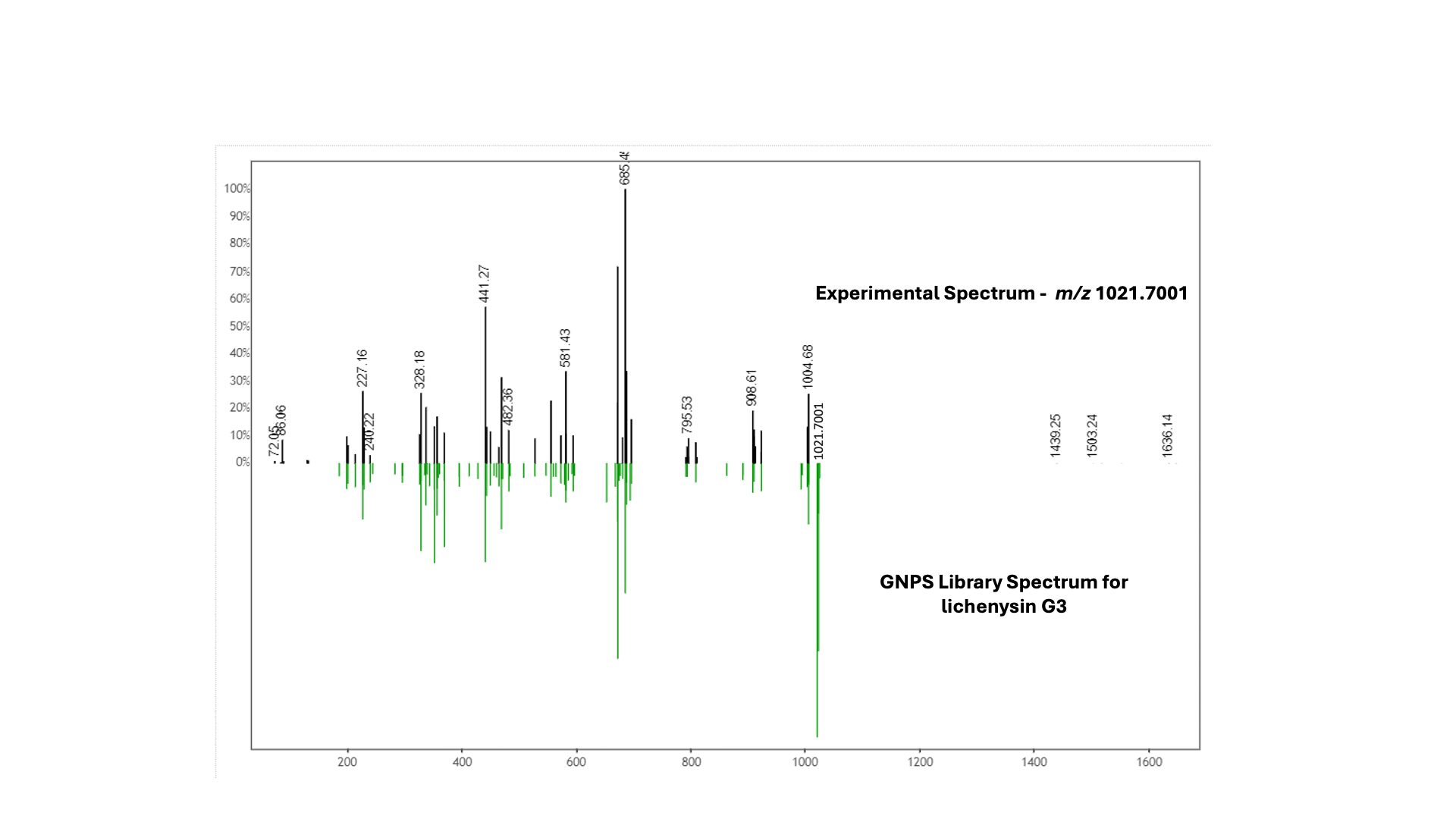


# **Figure S19.** GNPS mirror match plot for lichenysin G3 **(10)**. Green peaks represent the MS/MS library spectrum, while black peaks correspond to the MS/MS spectrum related to the ion *m/z* 1021.7001 acquired from the co-culture sample extract *Pseudonocardia* sp. ICBG 1050 x *Escovopsis* sp. ICBG 729. The comparison has a cosine score of 0.85. For **(10)** *m/z* calculated is 1021.6908 and error is 8.0 ppm.


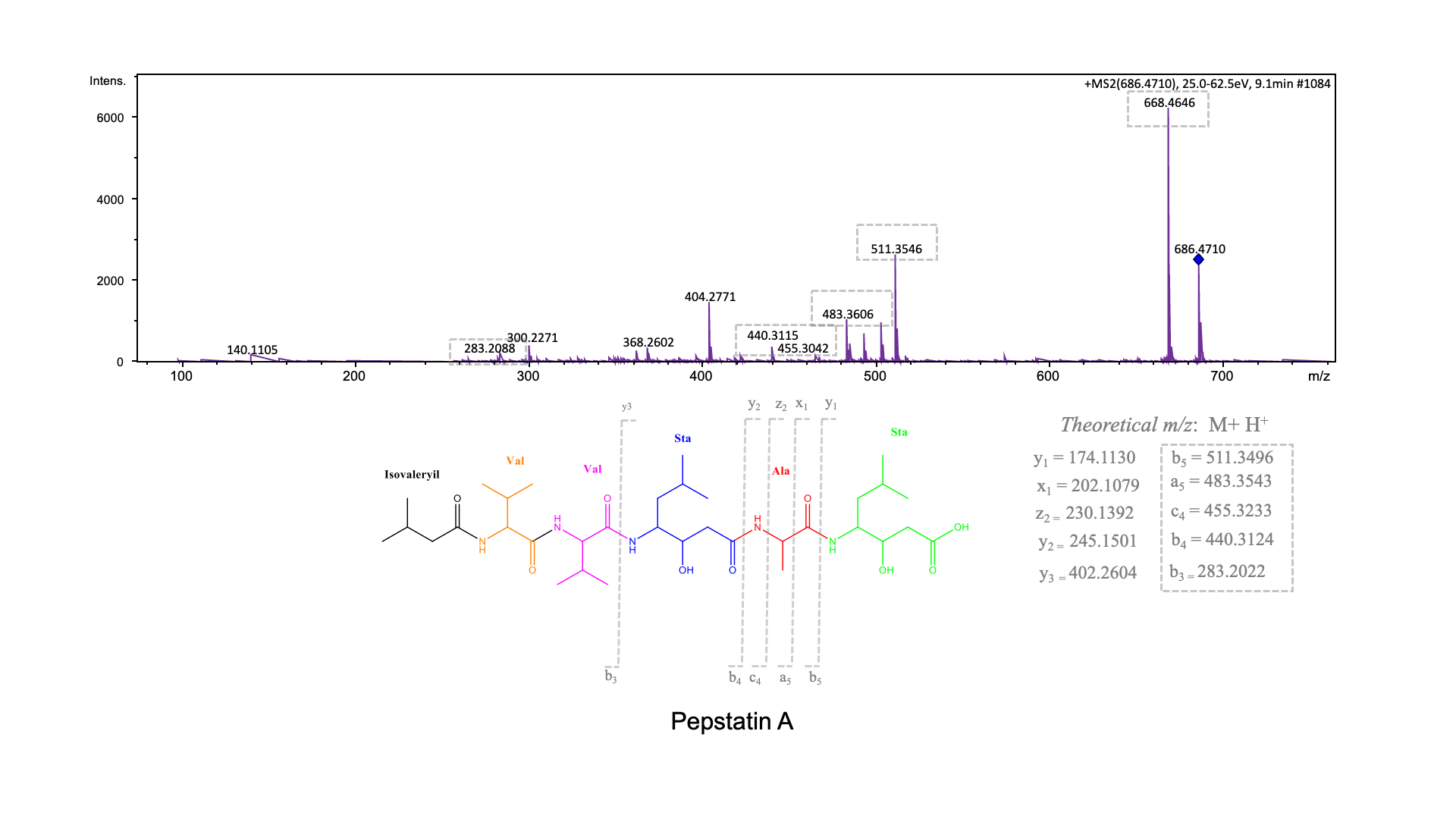
**Figure S20.** MS/MS fragmentation spectrum of pepstatin A (**11**) showing the major diagnostic b- and y-ions. MS² of *m/z* 686.4710 [M+H]⁺ reveals clear fragment ions corresponding to b₃–b₅, a₅, c₄, x₁, y₁–y₃, and z₂. The observed fragments match the predicted cleavage pattern for the Val–Val–Sta–Ala–Sta sequence, supporting the proposed linear peptide structure. This spectrum was acquired from the co-culture sample extract *Pseudonocardia* sp. ICBG 1245 x *Escovopsis* sp. ICBG 729. For **(11)** *m/z* calculated is 686.4699 and error is 1.6 ppm.


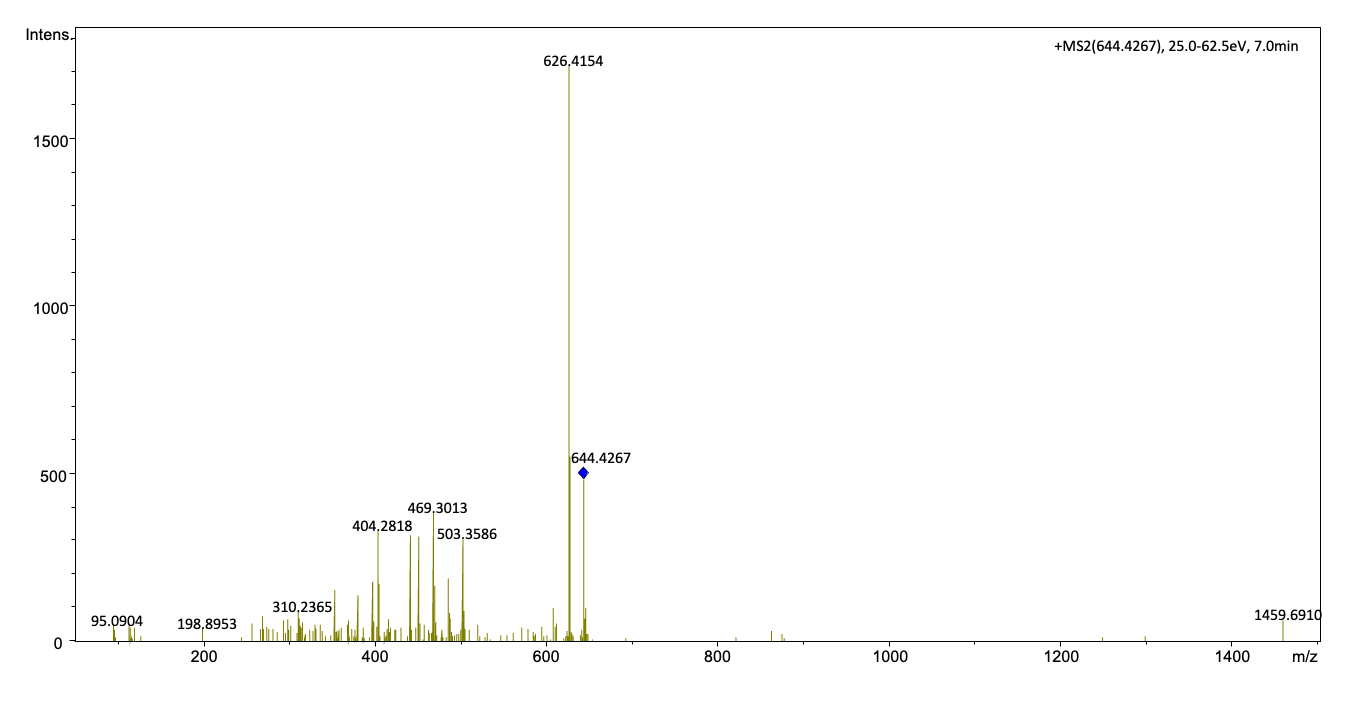


# **Figure S21.** HR-ESI-MS/MS spectrum of the [M+H]⁺ ion at *m/z* 644.4267 corresponding to pepstatin Ac **(12)** identified in the co-culture sample extract *Pseudonocardia* sp. ICBG 1245 x *Escovopsis* sp. ICBG 729. For **(12)** *m/z* calculated is 644.4229 and error is 5.9 ppm.


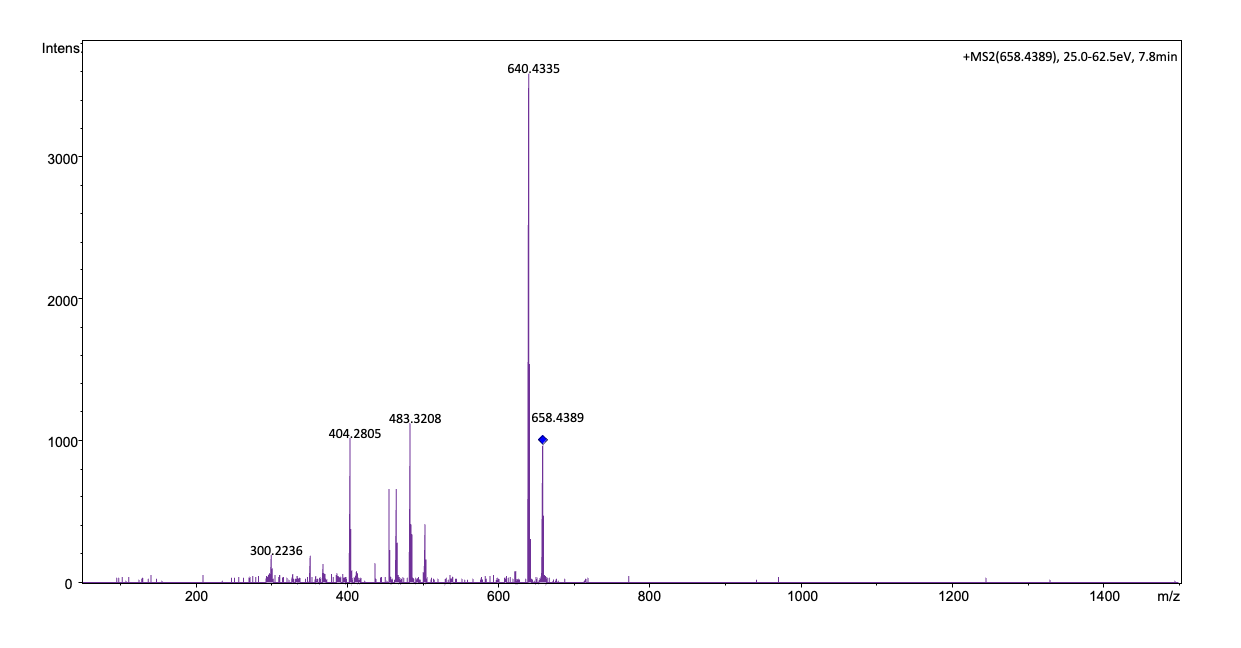


# **Figure S22.** HR-ESI-MS/MS spectrum of the [M+H]⁺ ion at *m/z* 658.4389 corresponding to pepstatin Pr **(13)** identified in the co-culture sample extract *Pseudonocardia* sp. ICBG 1245 x *Escovopsis* sp. ICBG 729.For **(13)** *m/z* calculated is 658.4386 and error is 0.5 ppm.

#


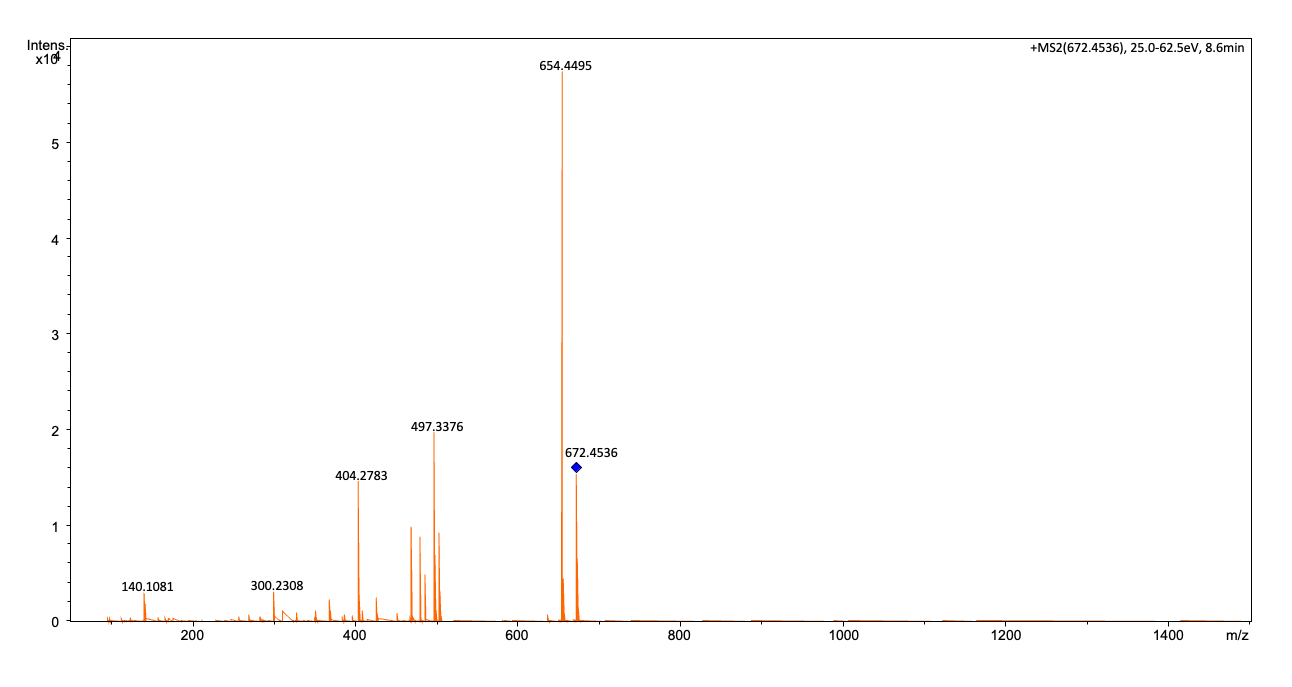


# **Figure S23.** HR-ESI-MS/MS spectrum of the [M+H]⁺ ion at *m/z* 672.4536 corresponding to pepsidin A **(14)** identified in the co-culture sample extract *Pseudonocardia* sp. ICBG 1245 x *Escovopsis* sp. ICBG 729. For **(14)** *m/z* calculated is 672.4542 and error is -0.9 ppm.


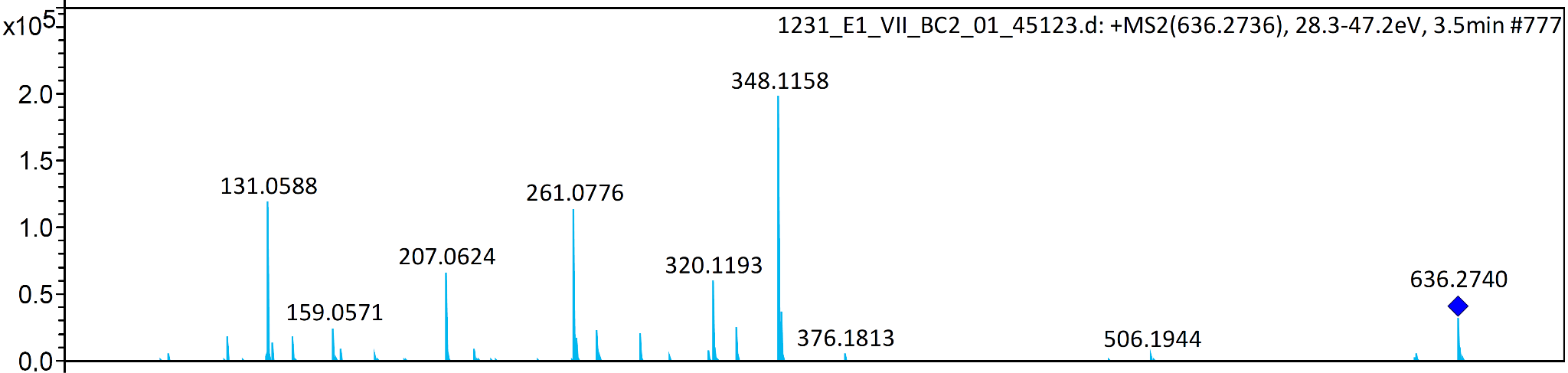


# **Figure S24.** HR-ESI-MS/MS spectrum of the [M+H]⁺ ion at *m/z* 636.2740 corresponding to attinimicin annotated in the co-culture sample extract *Pseudonocardia* sp. ICBG 1231 x *Escovopsis* sp. ICBG 729. These fragments were compared to attinimicin MS/MS fragments reported in literature (2).


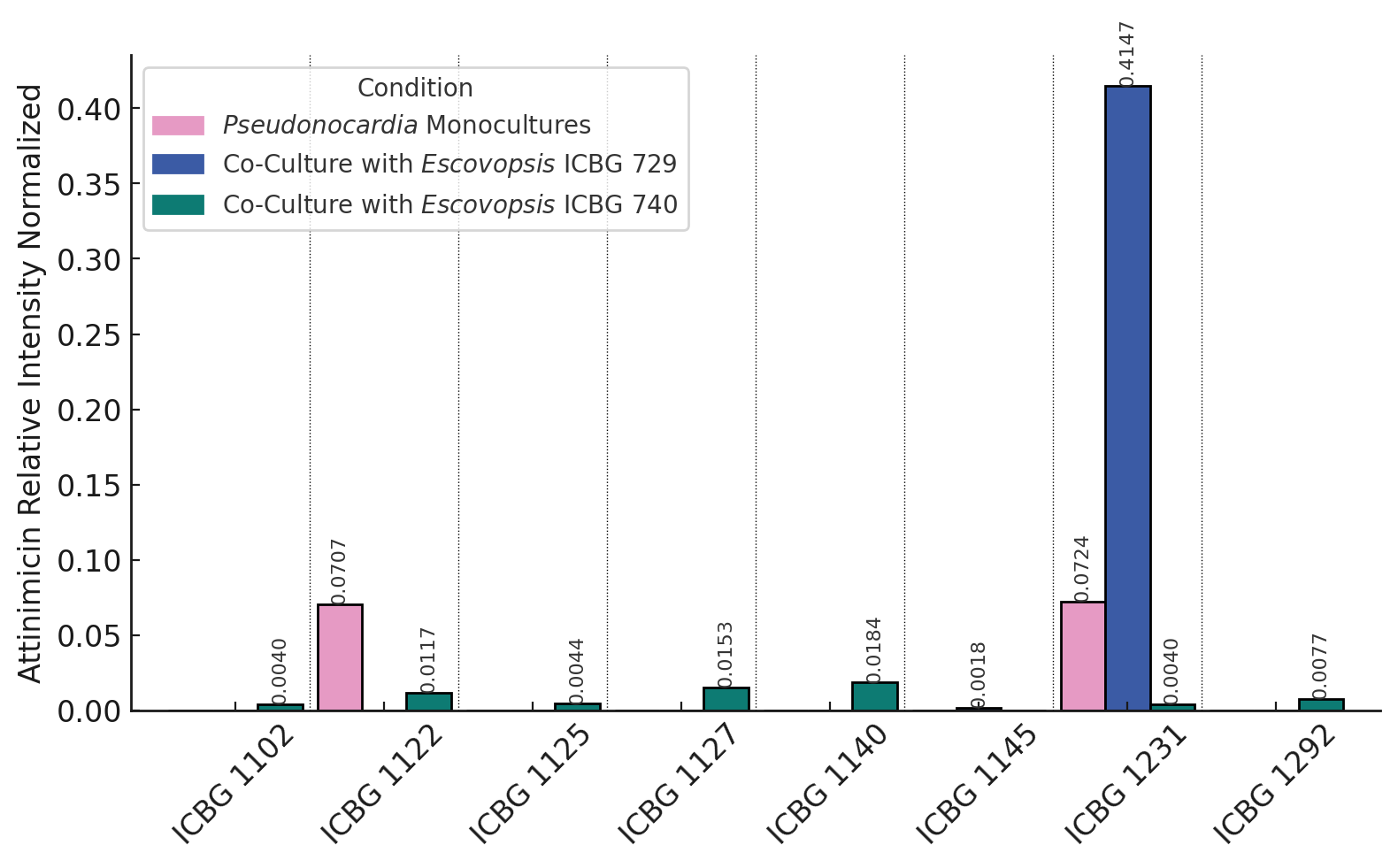


# **Figure S25.** Relative attinimicin MS detection across *Pseudonocardia* strains in monoculture and co-culture with two *Escovopsis* isolates. Normalized peak intensities of attinimicin (*m/z* 636.2740) are shown for each *Pseudonocardia* strain under monoculture conditions (pink) and during co-culture with *Escovopsis* ICBG 729 (blue) or ICBG 740 (green). Co-culture with *Escovopsis* ICBG 729 induced a marked increase in attinimicin production specifically in strain ICBG 1231. All peak intensities were normalized to the internal standard sulfamethazine (added at 2 µmol/L).

**
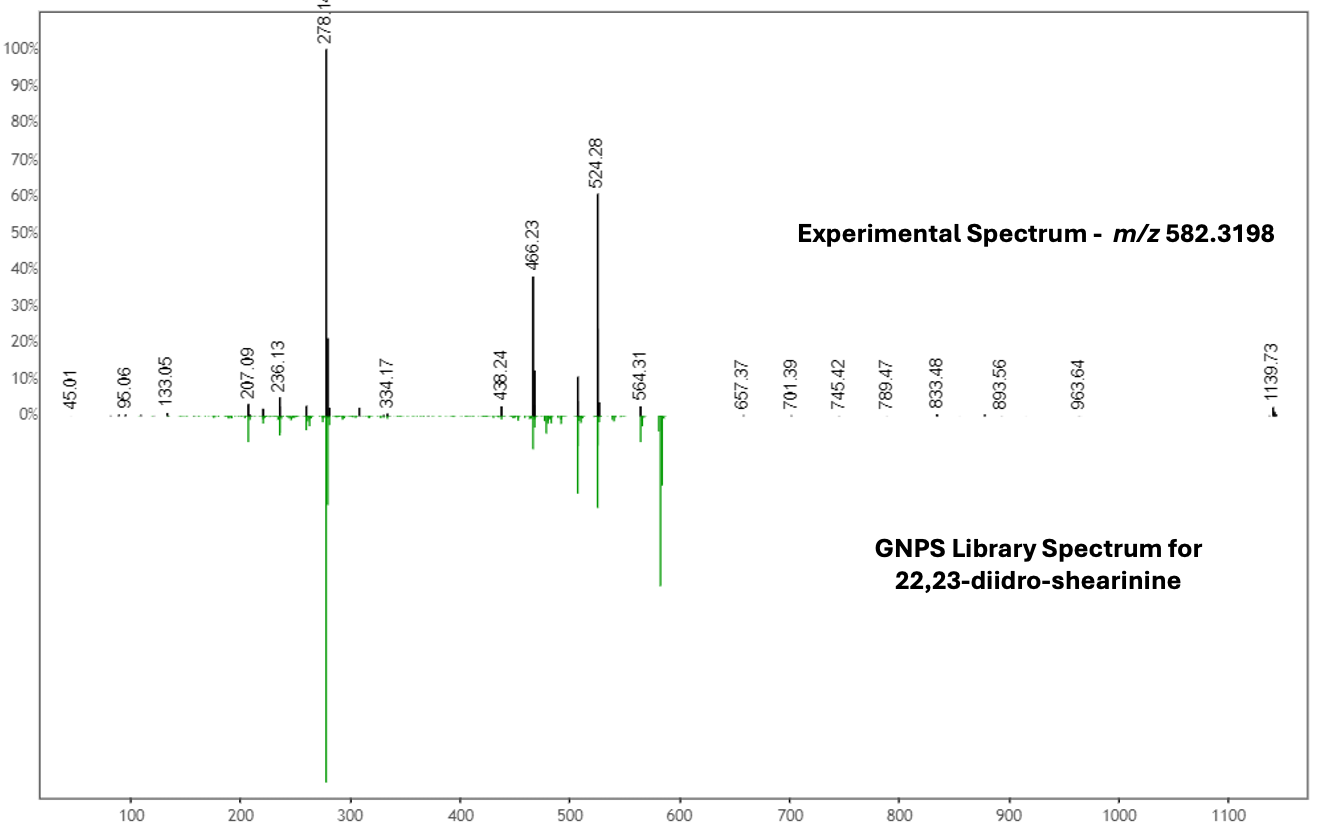
**

# **Figure S26.** GNPS mirror match plot for 22,23-dehydro-shearinine A (**15**). Green peaks represent the MS/MS library spectrum, while black peaks correspond to the MS/MS spectrum related to the ion *m/z* 582.3198 acquired from *Escovopsis* strains. The comparison has a cosine score of 0.89. For **(15)** *m/z* calculated is 582.3175 and error is -4.0 ppm.


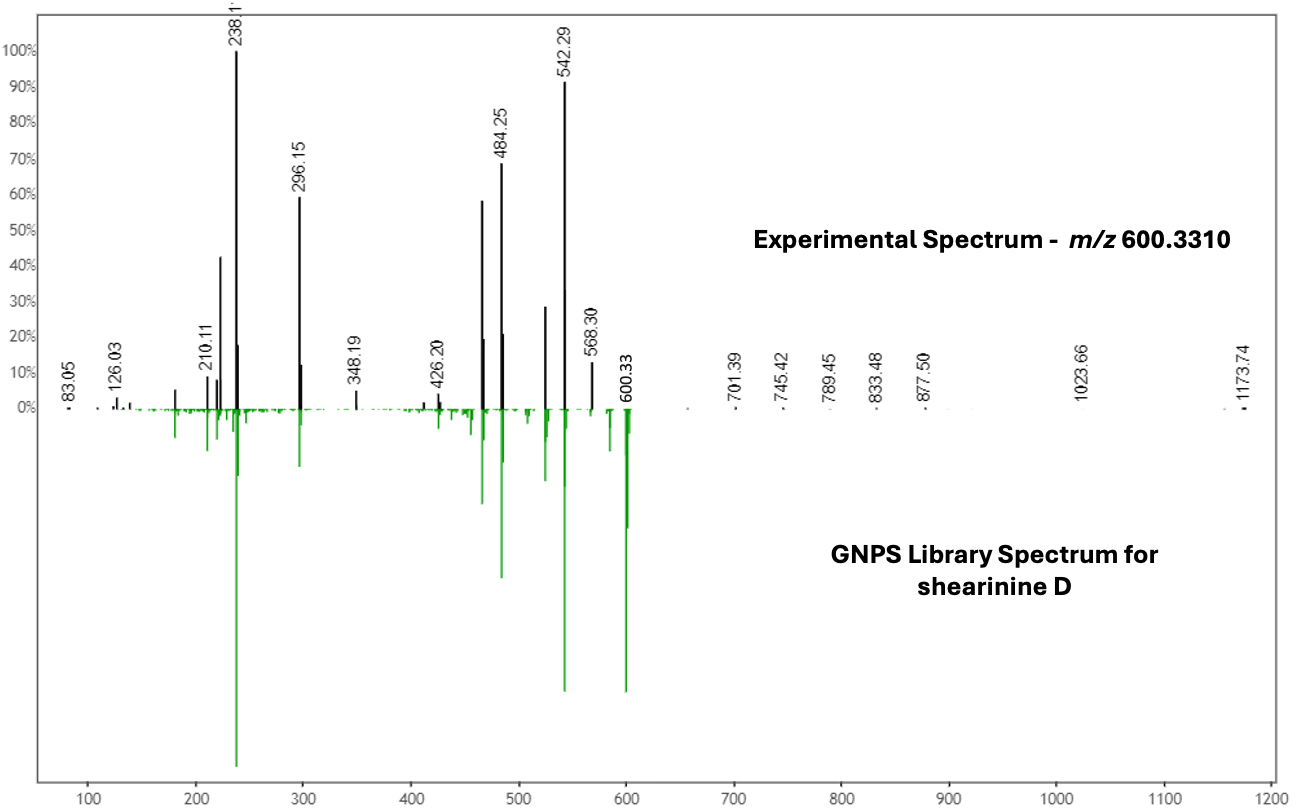


# **Figure S27.** GNPS mirror match plot for shearinine D (**16**). Green peaks represent the MS/MS library spectrum, while black peaks correspond to the MS/MS spectrum related to the ion *m/z* 600.3310 acquired from *Escovopsis* strains. The comparison has a cosine score of 0.86. For **(16)** *m/z* calculated is 600.3280 and error is 4.9 ppm.

**
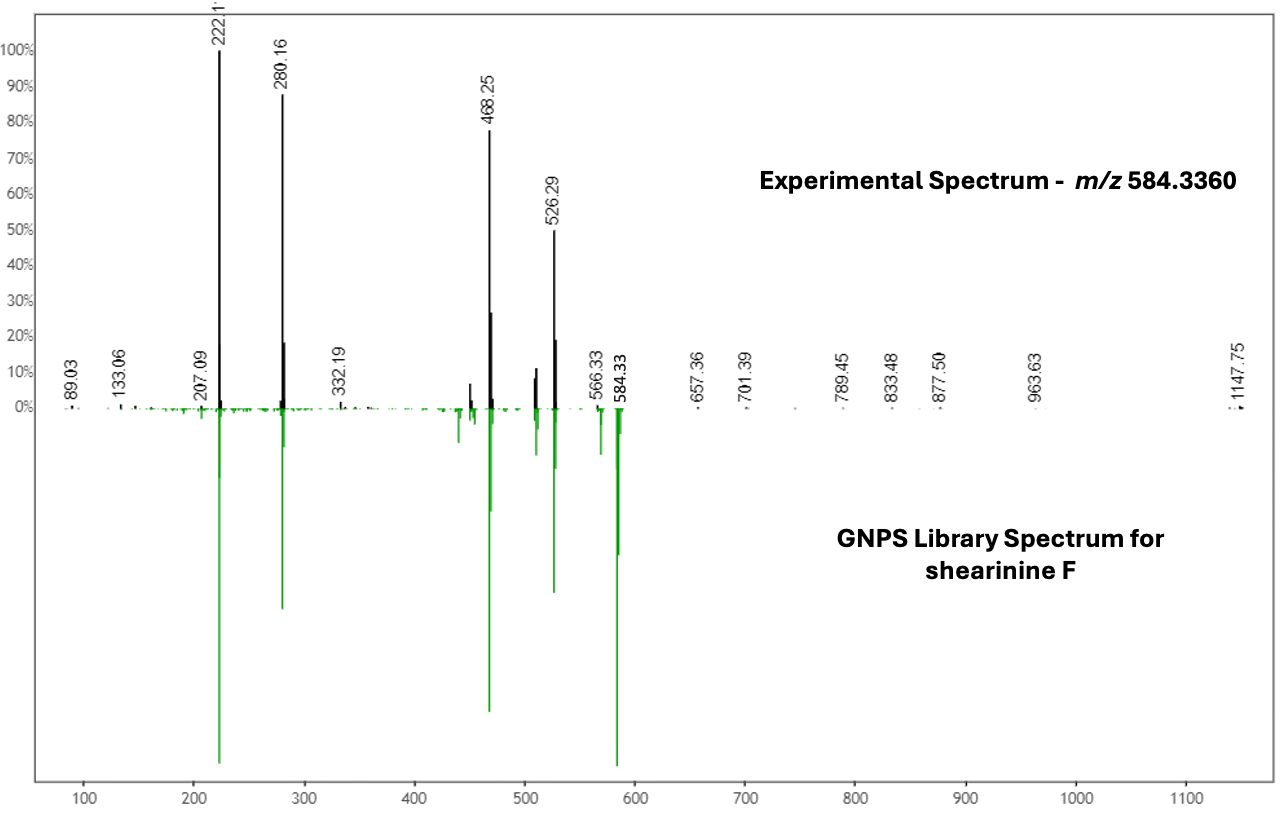
**

# **Figure S28.** GNPS mirror match plot for shearinine F (**17**). Green peaks represent the MS/MS library spectrum, while black peaks correspond to the MS/MS spectrum related to the ion *m/z* 584.3360 acquired from *Escovopsis* strains. The comparison has a cosine score of 0.94. For **(17)** *m/z* calculated is 584.3331 and error is 5.0 ppm.

**REFERENCES**

1. Bae M, Mevers E, Pishchany G, Whaley SG, Rock CO, Andes DR, Currie CR, Pupo MT, Clardy J. 2021. Chemical Exchanges between Multilateral Symbionts. Organic Letters https://doi.org/10.1021/acs.orglett.1c00068.

2. Fukuda TTH, Helfrich EJN, Mevers E, Melo WGP, van Arnam EB, Andes DR, Currie CR, Pupo MT, Clardy J. 2021. Specialized metabolites reveal evolutionary history and geographic dispersion of a multilateral symbiosis. ACS Central Science https://doi.org/10.1021/acscentsci.0c00978.
